# Unravelling the Structure of the Tetrahedral Metal-Binding Site in METP3 through an Experimental and Computational Approach

**DOI:** 10.3390/molecules26175221

**Published:** 2021-08-28

**Authors:** Salvatore La Gatta, Linda Leone, Ornella Maglio, Maria De Fenza, Flavia Nastri, Vincenzo Pavone, Marco Chino, Angela Lombardi

**Affiliations:** 1 Department of Chemical Sciences, University of Napoli Federico II, Via Cintia, 80126 Napoli, Italy; salvatore.lagatta@unina.it (S.L.G.); linda.leone@unina.it (L.L.); ornella.maglio@unina.it (O.M.); maria.defenza@unina.it (M.D.F.); flavia.nastri@unina.it (F.N.); vincenzo.pavone@unina.it (V.P.); 2 Istituto di Biostrutture e Bioimmagini (IBB), National Research Council (CNR), Via Mezzocannone 16, 80134 Napoli, Italy

**Keywords:** miniaturized proteins, tetrahedral metal binding site, metalloprotein models, spectroscopic characterization, NMR structure, bioinformatics

## Abstract

Understanding the structural determinants for metal ion coordination in metalloproteins is a fundamental issue for designing metal binding sites with predetermined geometry and activity. In order to achieve this, we report in this paper the design, synthesis and metal binding properties of METP3, a homodimer made up of a small peptide, which self assembles in the presence of tetrahedrally coordinating metal ions. METP3 was obtained through a redesign approach, starting from the previously developed METP molecule. The undecapeptide sequence of METP, which dimerizes to house a Cys_4_ tetrahedral binding site, was redesigned in order to accommodate a Cys_2_His_2_ site. The binding properties of METP3 were determined toward different metal ions. Successful assembly of METP3 with Co(II), Zn(II) and Cd(II), in the expected 2:1 stoichiometry and tetrahedral geometry was proven by UV-visible spectroscopy. CD measurements on both the free and metal-bound forms revealed that the metal coordination drives the peptide chain to fold into a turned conformation. Finally, NMR data of the Zn(II)-METP3 complex, together with a retrostructural analysis of the Cys-X-X-His motif in metalloproteins, allowed us to define the model structure. All the results establish the suitability of the short METP sequence for accommodating tetrahedral metal binding sites, regardless of the first coordination ligands.

## 1. Introduction

Metalloproteins employ a limited number of metal ions to accomplish countless different functions, including electron transfer, signal transduction and enzymatic catalysis [1,2,3]. To achieve this incredible variety, a wide array of protein sites has been selected over billions of years of evolution, each purposed towards a specific task [4]. In doing this, Nature has taken advantage of the unique physicochemical properties of metal ions, such as their small volume, Lewis acidity, coordination geometry preferences, specific ligand affinity, variable oxidation and spin states, which can be finely modulated by the protein matrix [5]. In fact, proteins and metal cofactors are in a synergic relationship, which enables metalloproteins to be both folded and functional [6]. The selection of the first coordination sphere ligands usually drives the selectivity toward a specific metal ion in a certain oxidation and spin state, thus controlling its reactivity [5]. Concurrently, the binding of a metal ion imposes structural constraints on the protein-derived ligands, which in turn adopt proper conformations to satisfy the metal coordination requirements. In some cases, protein folding occurs upon metal ion coordination, while in others, tight metal binding is assured by the protein, which imparts its own structural preferences to the metal ion [3]. Zinc-fingers are excellent examples of the first phenomenon, since the assembly of a tetrahedral Zn(II) binding site is essential to stabilize the global fold of the protein [7]. Conversely, in the blue copper proteins, a rigid protein environment dictates the coordination geometry of the metal ion [8]. The trigonal His_2_Cys coordination site observed in azurin is not optimal for either the Cu(II) or the Cu(I) oxidation states, and only minimal structural changes occur upon redox cycling. This “entatic” or “strained” state has the effect of enhancing electron transfer mediated by copper sites [9]. 

Owing to the limited collection of ligands available in protein scaffolds, the sole composition of the first coordination sphere could not account for the wide variability of functions observed among metalloproteins. Second-shell interactions strongly contribute to defining the specificity and reactivity of the metal-binding site, by orienting the first shell-ligands in the proper geometry and/or altering their electronic properties [10]. Several fundamental aspects of metal cofactor assembly and functions have been clarified by studying natural metalloproteins and their site-directed mutants [11,12]. In addition, numerous model systems have been developed by engineering metal-binding sites into artificial protein scaffolds [13,14,15,16,17,18,19,20]. They represent useful platforms for structure–activity relationship studies, allowing for a fast and easy screening of several mutants [21,22]. Furthermore, they are suitable candidates for biomedical, pharmaceutical or environmental applications [23,24,25,26,27]. Progress in this field has recently been accelerated by tremendous advances in computational methods and structural biology [28,29,30,31,32], and several mono-, di- or poly-nuclear metal binding sites have been successfully implemented into designed protein scaffolds [33,34,35,36,37,38,39]. 

One possible approach to metalloprotein design involves a miniaturization process. It starts with the analysis of a metalloprotein three-dimensional structure and aims to detect the lowest number of interactions needed to rebuild the metal binding site out of the whole protein [40]. The presence of geometric relationships between the elements of secondary structure that define the active site aids the design process, since the target structure can be generated by applying symmetry operations to a minimal fragment. We have successfully applied this approach to the development of miniaturized metalloproteins [41,42,43,44,45,46,47]. In particular, the retrostructural analysis carried out on rubredoxin (Rds) has allowed us to develop METP [48], made up of a β-hairpin undecapeptide with two properly spaced Cys residues, that self-assembles in the presence of a coordinating metal ion, to form a C_2_ symmetric tetrathiolate binding site. The characterization by several spectroscopic techniques showed that METP retains the main features of the parent protein. It binds iron, closely resembling the rubredoxin active site spectroscopic features. Encouraged by these results, we focused our attention on the development of METP analogues. By systematically varying the number and chemical nature of the ligands, our work was aimed at verifying the feasibility of METP to serve as a general scaffold for engineering tetrahedral metal-binding sites. Even though tetrahedral binding sites have been implanted into de novo designed proteins [48,49,50,51,52], METP holds the advantage of being the smallest scaffold reported to date.

Here, we report the design, synthesis, spectroscopic and structural characterization of METP3, a new analogue conceived to reproduce a Cys_2_His_2_ metal binding site. Only small changes in the undecapeptide sequence of METP were needed for preserving the tetrahedral geometry of the metal site upon modification of the first-coordination sphere ligands. The correctness of the design was proven by solution spectroscopic characterization. A survey of zinc-containing metalloprotein structures in the PDB was also performed, enabling the dimeric structure of Zn(II)-METP3 to be deciphered, thus offering new insights for subsequent design.

## 2. Results

### 2.1. Design

**Figure 1:**
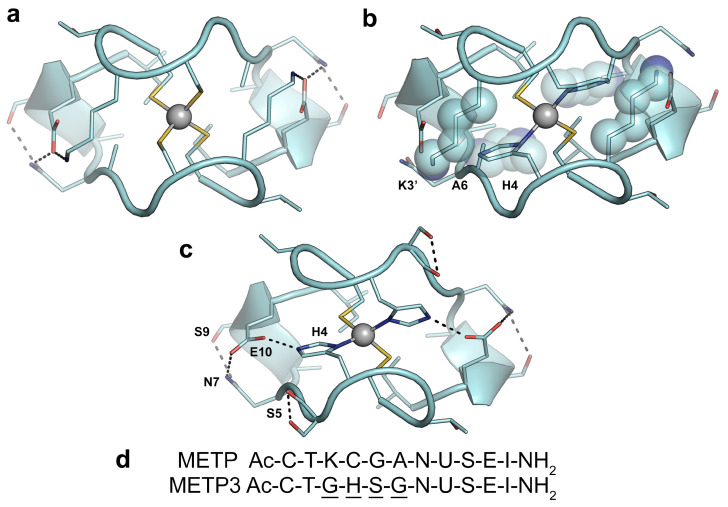
Redesign of the Cys_4_ METP binding site into Cys_2_His_2_. (**a**) Energy minimized structure of Zn(II)-METP complex. The coordinating and hydrogen bonded residues are shown as sticks. (**b**) Cys_2_His_2_ Zn(II)-binding site housed in the unoptimized Zn(II)-METP model structure. The steric clashes of His^4^ with Lys^3’^ and Ala^6^ side chains are evidenced as spheres. (**c**) Energy-minimized model of Zn(II)-METP3 complex. In all the panels, the Zn(II) ion is reported as a gray sphere. Peptide backbone is represented as a ribbon. (**d**) Comparison of the monomeric peptide sequence of METP and METP3. U represents 2-aminoisobutyric acid (Aib).

The design of METP3 peptide was based on the previously characterized METP prototype [48]. To convert the Cys_4_ (Figure 1a) into a Cys_2_His_2_ (Figure 1c) metal binding site, the Cys residue at position four in each METP chain was mutated to His (Figure 1b). Additional substitutions were necessary to complement the changes in the first coordination sphere. Indeed, Cys^4^His substitution resulted in clashes of His^4^ with Lys^3’^ and Ala^6^ side chains (Figure 1b). To accommodate the larger His ligand into the site, Lys^3^ and Ala^6^ were replaced by Gly. These substitutions altered the pattern of the inter-chain hydrogen bond interactions with respect to the prototype model, where Lys^3(3’)^ of one peptide chain was hydrogen bonded to Glu^10’(10)^ of the other. Interestingly, Lys^3^Gly substitution provided the appropriate distance for a second-shell-hydrogen bond between His^4(4’)^ N and Glu^10’(10)^, which could stabilize the metal binding geometry (Figure 1c). The hydrogen bonds between Glu^10’(10)^ and Asn^7(7’)^ residues are conserved as in the parent model. Moreover, Gly^5(5’)^ of METP was replaced with a Ser, whose side chain forms a hydrogen bond with the carbonyl backbone of itself and may also improve water solubility of the complex. This extended hydrogen bond network, together with hydrophobic Ile^11^-Ile^11’^ interaction, is expected to drive the homodimerization of METP3. Furthermore, such interchain interactions are expected to stabilize only a single topology of the dimer. Symmetrical dimerization (via a C_2_ axis) in the presence of a tetrahedrally coordinating metal ion may in principle give rise to Δ and Λ enantiomers. They are actually diastereomers because of the chirality of the amino acids [48]. As in the case of METP, the designed interactions may stabilize a Λ configuration, as found in natural Rds [48]. The amino acid sequences of the newly designed peptide METP3 and of the prototype METP are reported in Figure 1d.

### 2.2. Synthesis

The monomeric MEPT3 peptide was obtained in good yield (90%) by solid-phase synthesis, using standard Fmoc/tBu protocols. The crude peptide was first reduced and purified by preparative RP-HPLC (purity > 95%). The homogeneity and identity of the final product was confirmed by analytical RP-HPLC and MALDI-TOF mass spectrometry (Figures S1 and S2).

### 2.3. Metal Binding Properties

METP3 metal binding properties were studied in the presence of different divalent metal ions, in particular cobalt, zinc and cadmium ions. 

**Figure 2:**
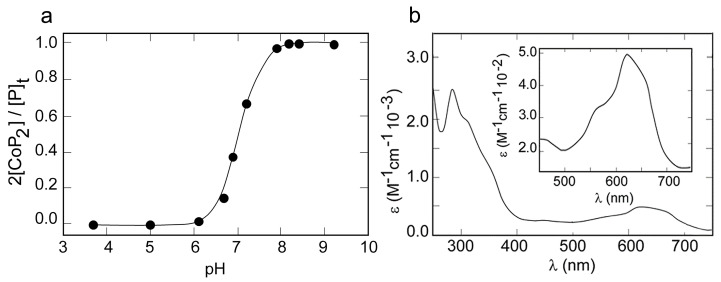
(**a**) pH titration of Co(II)-METP3 complex in the presence of 10% excess of Co(II). (**b**) UV-visible absorption spectrum of Co(II)-METP3 complex (10 mM TRIS buffer pH 8.2).

Cobalt was selected for initial binding studies, because Co(II) complexes show distinctive UV-visible spectra, which are sensitive to the metal coordination geometry and to the nature of the ligands [53]. To determine the optimal pH conditions for coordination, a UV-vis pH titration of METP3 in the presence of Co(II) ions was performed in the pH range 3.8–9.2 (Figure 2a). An increase in the absorption band intensities was observed upon raising the pH from 6 to 8.2. This indicates ligands deprotonation and concomitant coordination, with a midpoint transition at pH 7.1. Based on these results, all the spectroscopic studies were performed at pH 8.2.

Figure 2b shows the UV-visible absorption spectrum obtained upon addition of a 10% molar excess of CoCl_2_ to the fully reduced peptide in aqueous solution.

Table 1 compares the UV-visible spectral data of the Co(II) complexes of METP3 and METP [48]. The spectral data of Co(II)-GGG, a minimal peptide model of a zinc finger protein [54], and of Co(II)-CP-1, a 26 amino acid peptide based on the consensus sequence of 131 zinc finger domains [55], containing a Cys_2_His_2_ and a Cys_3_His coordination site, respectively, are also reported for comparison.

The spectrum of Co(II)-METP3 shows an intense absorption band in the near-ultraviolet at 308 nm, and an absorption envelope in the visible region (500–700 nm). The former band can be assigned to S→Co(II) ligand-to-metal charge-transfer (LMCT) transitions, indicative of cysteine coordination [49]. The observed molar absorptivity (ε_308 nm_) value of 2100 M^−1^cm^−1^ indicates the presence of two thiolate ligands in the first coordination sphere, in agreement with the average value of ≈ 900–1200 M^−1^ cm^−1^ per thiolate-cobalt bond, as reported in the literature [56].

The position and intensity of the *d-d* electronic transitions in the visible region give further insights into the Co(II) coordination environment. The absorption envelope displays a maximum at 624 nm (ε = 470 M^−1^ cm^−1^) and shoulders at 649 nm (ε = 440 M^−1^ cm^−1^) and at 564 nm (ε = 313 M^−1^ cm^−1^). The intensities of these bands (ε ≥ 300 M^−1^ cm^−1^) are distinctive of a tetrahedral or distorted tetrahedral geometry [57] and rule out any higher coordination number. Indeed, lower molar absorptivity values are expected for both pentacoordinate and octahedral Co(II) sites [53,55,58]. The energy of *d-d* transitions is also sensitive to the number of thiolate ligands. Based on the position of imidazole and thiolate in the spectrochemical series, the tetrahedral ligand field splitting (Δ_t_) increases almost linearly as thiolates are replaced by imidazoles [59], with a consequent shift of the *d-d* transition at higher energies. In agreement with these observations, a hypsocromic shift of all the absorption bands is observed when comparing the spectral data of Co(II)-METP3 with those of Co(II)-METP (Table 1). Moreover, the spectral features of Co(II)-METP3 are also consistent with those reported in the literature for Co(II)-Cys_2_His_2_ binding sites in proteins and model systems [54,59]. 

**Figure 3:**
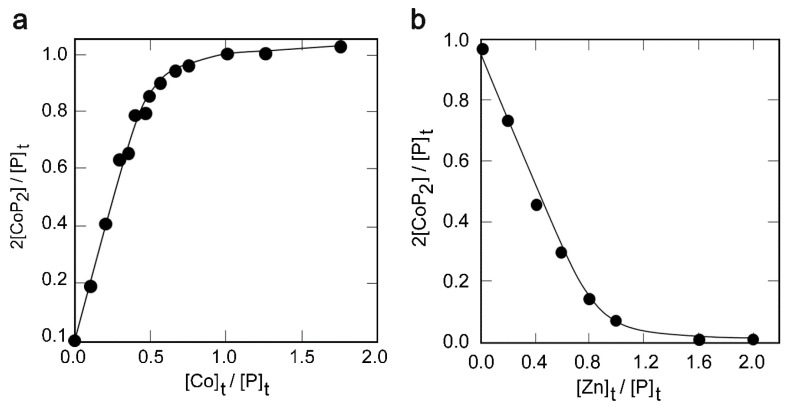
(**a**) Spectrophotometric titration of METP3 peptide with Co(II). The saturation fraction 2[CoP_2_]/[P]_t_ is plotted against the concentration ratio [Co]_t_/[P]_t_. Solid line shows the best fit of data points to Equation (1). (**b**) Spectrophotometric competition experiment of Co(II)-METP3 complex with Zn(II). The saturation fraction 2[CoP_2_]/[P]_t_ is plotted against the concentration ratio [Zn]_t_/[P]_t_. Solid line shows the best fit of data points to Equation (2).

The stoichiometry of the Co(II)-METP3 complex formation and the dissociation constant (K_D_) were determined by direct spectrophotometric titration. The saturation fraction 2[CoP_2_]/[P]_t_ was plotted against the concentration ratio [Co]_t_/[P]_t_ (Figure 3a) and the experimental data were fitted to a binding isotherm (see experimental section, Equation (1)). The best fit gave a peptide:Co(II) ratio of 2:1 and a K_D_ of (85 ± 4) µM.

Zn(II) binding to METP3 was investigated by competition experiments, because Zn(II) complexes are spectroscopically silent. Zn(II) addition to a solution of the peptide containing 3.5 eq. of Co(II) led to a sharp decrease in the UV-visible bands of the cobalt complex (Figure S3), indicating that Zn(II) readily displaces Co(II). In this condition, a K_D_ value of (5.6 ± 0.2) µM was obtained for Zn(II) binding to METP3 (Figure 3b), proving that METP3 binds Zn(II) more tightly than Co(II).

Similar competition experiments were also performed with Cd(II), in the same experimental conditions used for Zn(II). Displacement of Co(II) with Cd(II) resulted in fading of the visible bands of the Co(II)-METP3 complex (Figure S4) and a K_D_ of (5.0 ± 1.0) µM was determined for the cadmium complex.

Analogously to METP [48], the observed lower affinity of METP3 peptide for Co(II), with respect to Zn(II) and Cd(II) (Table 2), is fully consistent with a tetrahedral coordination site [59]. In addition, the comparable affinity of METP3 toward Cd(II) and Zn(II) is expected because of the same metal *d*^10^ configuration. 

Coordination of metal ions to the METP3 binding site involves deprotonation of the two cysteine to thiolate. Therefore, undesired oxidation products could be formed upon metal complex dissociation, via disulfide bond formation. RP-HPLC analyses were performed to investigate the stability of METP3 metal complexes towards Cys oxidation. A reference sample of the intermolecular oxidized form, corresponding to the disulfide bridged dimer, was prepared by oxidation of the fully reduced pure peptide with DMSO (see experimental and Figure S5). The reduced monomeric peptide METP3 and the dimeric intermolecular oxidized form display different retention times (Figures S1 and S5), thus being easily distinguishable by HPLC analysis. 

Interestingly, RP-HPLC analysis of a Zn(II)-METP3 solution, performed 4 days after the preparation, revealed the peptide chain to be in the almost fully reduced state (main peak at Rt = 13.9 min, Figure S6a), with only a small percentage of the intermolecular oxidized product (Rt = 15.6 min, Figure S6a). Conversely, the peptide was almost fully oxidized (87%) only 2 hours after the preparation of the Co(II)-METP3 complex (Figure S6b). These results highlight that the higher affinity of METP3 toward Zn(II) with respect to Co(II) correlates with a lower propensity of the Cys residues to form disulfide bridge.

### 2.4. CD Spectroscopy

**Figure 4:**
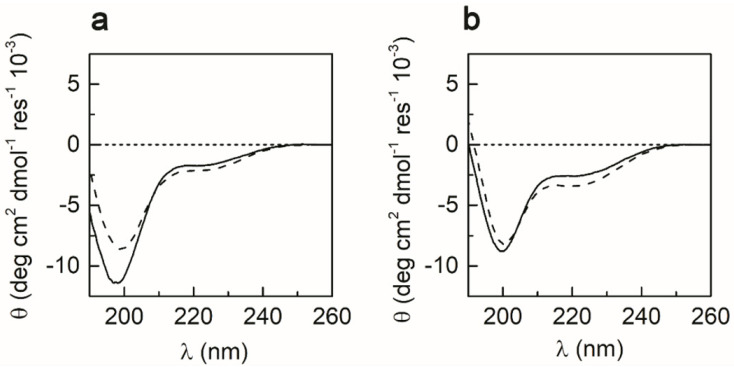
Far-UV CD spectra at 0.2 mM peptide concentration of fully reduced METP3 (solid lines) and Zn(II)-METP3 complex (dashed lines) in: (**a**) H_2_O at pH 7.5 or (**b**) TFE.

The effects of the solvent and metal binding on METP3 folding were examined by CD spectroscopy in the far UV region (Figure 4). The CD spectrum of the fully reduced peptide (0.2 mM) in aqueous solution indicates an unfolded peptide [60], exhibiting a strong negative band at 198 nm and a small shoulder at 220 nm (Figure 4a). The coordination of Zn(II) causes a decrease in the intensity of the minimum at 198 nm, together with a slight increase in the minimum at 220 nm. CD spectra of apo- and Zn(II)-METP3 were also acquired in 2,2,2-trifluoroethanol (TFE), due to the stabilizing effect of this solvent on both α and β folds [61]. In the spectrum of apo-METP3 in TFE (Figure 4b), the lower wavelength minimum appears less intense and slightly red-shifted (200 nm) with respect to the spectrum recorded in aqueous solution (198 nm). A slight decrease in the intensity of the minimum at 200 nm, with a concomitant increase in the minimum at 220 nm is observed upon Zn(II) binding. The spectral changes observed upon metal binding indicate that the Zn(II) coordination induces structural stabilization, both in aqueous and TFE solutions [60]. However, the peptide chain is better folded in TFE, where it assumes a turned conformation even in the apo state.

### 2.5. NMR Analysis

The solution structure of Zn(II)-METP3 was investigated by NMR spectroscopy. A complete characterization in aqueous solution was hampered by the rapid exchange of the amide protons with the solvent under the alkaline conditions required for metal binding. Different solvent systems were explored for the NMR analysis, including methanol, as used for the prototype METP [48]. Among them, TFE was selected as the best solvent, as ascertained by CD spectroscopy. The use of TFE, compared to methanol, allowed for better spectral resolution and subsequent peak assignment. 

**Figure 5:**
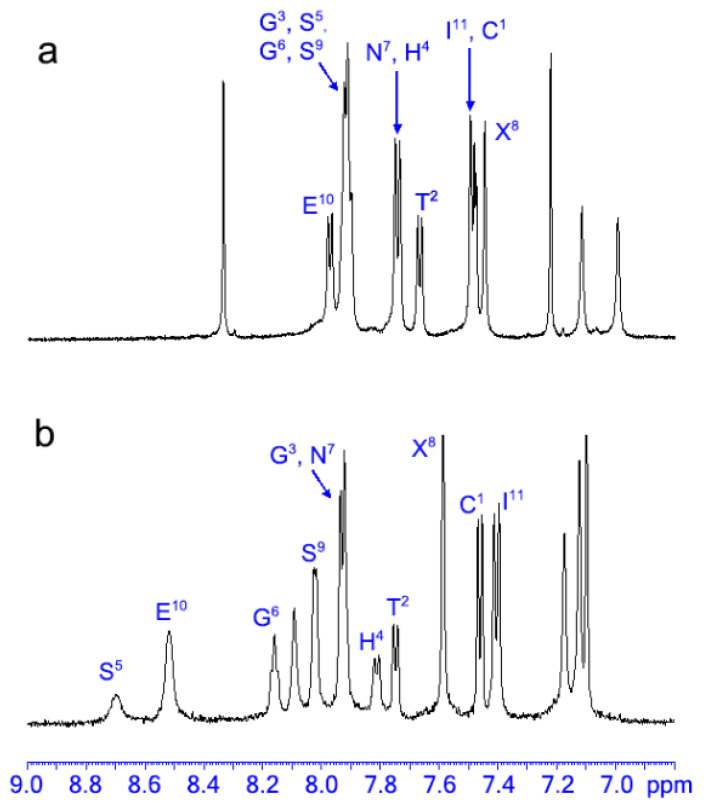
Amide proton region of the 1D ^1^H NMR spectra of: (**a**) fully reduced METP3 (**b**) Zn(II)-METP3 complex. Both samples were prepared in CF_3_CD_2_OH.

First, 1D ^1^H NMR spectra were recorded to qualitatively ascertain the extent of structural organization of the molecule in solution, both in the presence and in the absence of Zn(II). Figure 5 shows the amide regions of ^1^H NMR spectra of the fully reduced apo-METP3 and of the Zn(II)-METP3 complex in deuterated TFE (CF_3_CD_2_OH).

In the absence of Zn(II), the ^1^H NMR spectrum of the peptide shows poor peak dispersion and severe resonance overlap in the amide region, suggesting the absence of a stable conformation (Figure 5a). Upon addition of 1.1 equivalents of Zn(II) to the peptide, the amide proton resonances become well-dispersed, indicative of a well-defined structure (Figure 5b). Furthermore, a single set of resonances was observed for each residue, clearly indicating that the complex is a symmetric dimer.

**Figure 6:**
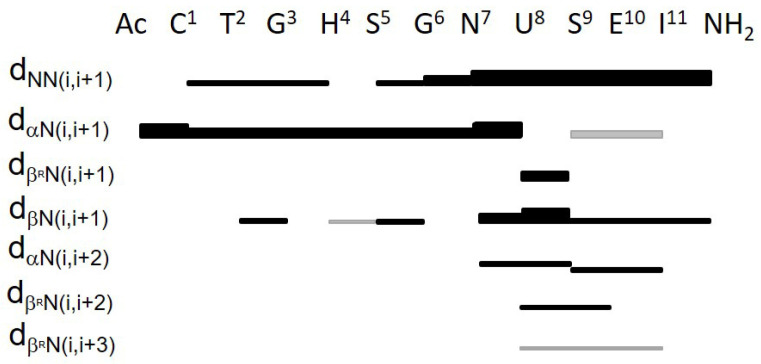
Summary of short- and medium-range NOE connectivities observed for Zn(II)-METP3 complex. The thickness of solid bars indicates the relative intensity of the NOEs, i.e., weak, medium and strong. Grey bars indicate spectral overlap.

Additionally, 2D NMR experiments (TOCSY and NOESY) were performed to obtain structural information. Examination of Nuclear Overhauser Effect (NOE) effects, temperature coefficient and ^3^J_NH-αCH_ coupling constant values allowed us to delineate METP3 secondary structure. A total of 10 restraints per residue were found, and only few NOEs were observed in the segment spanning residues 5–7. Figure 6 summarizes the sequential and medium-range NOE connectivities.

The presence and the relative intensities of the NH_i_-NH_i+1_ NOE connectivities in the Asn^7^-Glu^10^ segment suggest an incipient 3_10_-helix, characterized by a type III β-turn enclosing Aib^8^-Ser^9^ residues. The turned conformation is further supported by the presence of βCH_i_-NH_i+1_, βCH_i_-NH_i+2_ medium range contacts together with weaker NOE effects αCH_i_-NH_i+2_. The presence of NOE contacts between Asn^7^-αCH and Ser^9^-NH, Ser^9^-αCH and Ile^11^-NH, Aib^8^-βCH_3_^proR^ and Glu^10^-NH (see Figure 6), and the concomitant absence of αCH_i_-NH_i+4_ (d_αN_(i, i + 4)~5.5 Å for 3_10_ helix) also support this conformation [62,63]. The ^3^J_NH-αCH_ coupling constant value of Ser^9^ (4.2 Hz) (Table S1) is very close to the theoretical value expected for a corner residue (position i+2) of a type III β-turn [63].

In the N-terminal region, the presence of NH_i_-NH_i+1_ connectivities suggests an α-turn conformation in the Cys^1^-Ser^5^ segment. An extended conformation can be inferred in the Gly^6^-Asn^7^ segment, because the ^3^J_NH-αCH_ coupling constant values were found as 8.2 and 7.7 Hz for Gly^6^ and Asn^7^, respectively, and the ϕ solution of the Karplus equation [64] was −180° for both residues. 

The conformational analysis carried out on this molecule is in striking agreement with the secondary structure elements of the designed METP3 model. The latter was thus used as a template to construct a plausible initial model of the monomeric peptide chain, that was subsequently subjected to restrained molecular dynamic (RMD) simulation, using the experimental NOE contacts [65]. Figure S7 shows the backbone superposition of an ensemble of 30 representative NMR conformers along the RMD trajectory. The root mean square deviation (RMSD), derived from the backbone atom superposition between each member of the ensemble and the average structure, is (1.5 ± 0.2) Å.

In all structures, the N-terminal region (Cys^1^-His^4^) adopts an α-turned conformation, and the C-terminal segment can be described as a single 3_10_ helical turn. The RMSDs for the backbone atom superposition are (0.5 ± 0.1) Å and (0.4 ± 0.1) Å, for the N- and C-terminal segment, respectively (Figure S8). These two segments display a considerable conformational similarity with the designed monomer. The RMSDs, obtained from the N- and C-segment backbone atom superposition of the average RMD simulation derived model and the designed model, are (0.8 ± 0.2) Å and (0.3 ± 0.2) Å, respectively. 

The linker segment (Ser^5^-Gly^6^) displays a higher conformational freedom, preventing the N- and C-terminal regions from being uniquely oriented. Furthermore, only one connectivity can be assigned as inter-chain. In fact, a careful analysis of the NOESY spectrum showed the presence of a NOE involving the ε^1^ and amide protons of His^4(4’)^ (8.10/7.92 ppm) (Figure S9). Such contact can only be assigned as an inter-chain correlation, because these two histidine protons are far away from each other in the monomeric structure (d > 6 Å), but could be close in space, when the two monomers would be arranged in the homodimer. Additional inter-chain connectivities, needed to unequivocally define the correct positioning of the two monomers and to delineate the overall topology of the homodimer were not observed in the explored experimental conditions. Thus, a retrostructural analysis was performed for deciphering the structure of the Zn(II)-METP3 complex.

### 2.6. Retrostructural Analysis of Pseudo-Symmetric Dimers Containing CXXHX Motif

To help to solve the dimeric structure, compatible with the experimental NMR data, a search for pseudo-symmetric dimers, containing at least one CXXHX coordination environment, was carried out in the Protein Data Bank (PDB). 

To create an unbiased data set, a detailed analysis of the segment sequence and conformation was performed. Similar structures were removed from the initial database (see experimental section), obtaining a set of 129 structures (out of ≈2100 protein structures deposited in the PDB that satisfy the initial filters). Furthermore, only those containing the Zn binding-site in a C-X_1_-X_2_-H-X_3_ sequence with an α-turn conformation were manually selected (Table S2). 

**Figure 7:**
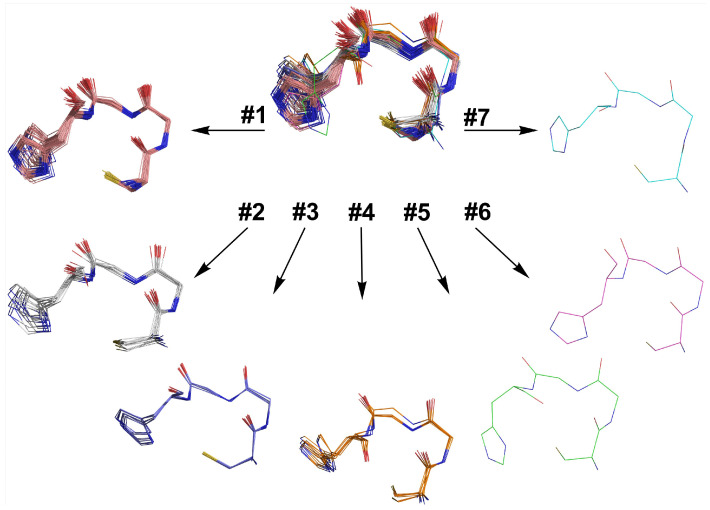
Superimposed C-X_1_-X_2_-H-X_3_ motifs as obtained from the clusterization procedure.

The Zn-sites were clustered according to their structural similarity, as measured by RMSD. This process resulted in four clusters and three outlier structures that could not be attributed to any clusters within the RMSD cutoff (Figure 7). An overall average RMSD of 0.5 Å and a maximum RMSD of 1.0 Å were observed, whereas the highest averaged distance, observed between any given centroid and the members of its cluster, was 0.3 Å (clusters 2 and 4).

The sequence motif did not appear to be related to a specific function, as more than 16 different functions have been assigned to the evaluated proteins (Figure S10). A first analysis of the primary structure of the non-redundant data set (Table S3) did not show prominent residue preferences for positions X_1_, X_2_ and X_3_ (Table 3 and Figure S11). 

However, inspection of Table S3 shows that: (i) Pro residue is highly recurrent in position X_1_ in clusters 2 and 3, whereas it is never found in this position in clusters 1 and 4 with only one exception; (ii) Gly residue is never found in position X_1_ and X_2_, while it is distinctive of position X_3_ in cluster 2; (iii) Thr is very frequent in position X_3_ in cluster 1, as it is involved into a C-capping hydrogen bond.

**Figure 8:**
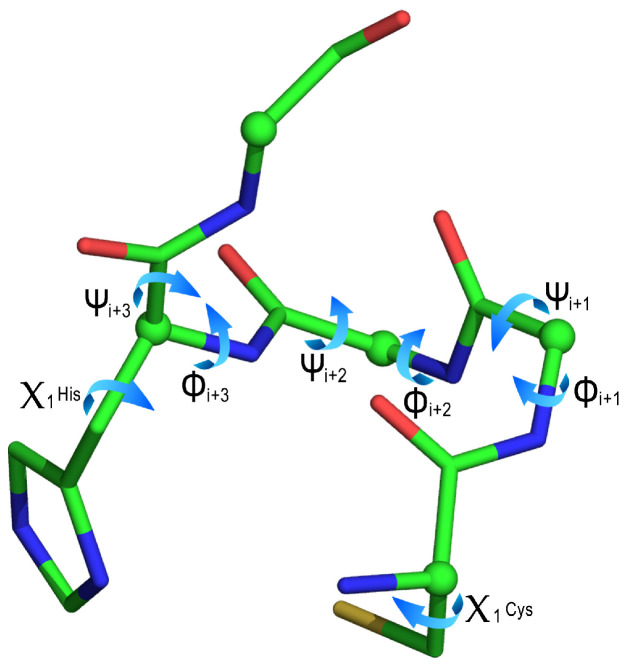
Prototypical C-X_1_-X_2_-H-X_3_ motif, describing the ϕ, ψ and χ_1_ dihedral angles.

A detailed analysis of the C-X_1_-X_2_-H-X_3_ backbone torsion angles (ϕ and ψ), and of Cys and His χ_1_ dihedral angles was performed for highlighting differences among the clusters (Figure 8 and Table 4).

In particular, the analysis carried out on main chain torsion angles confirmed that the selected motif adopted an α-turn conformation (type Iα_RS_) in all the clusters [67,68]. Few structures did not show the expected i, I + 4 hydrogen bond (seven of them possessing a Pro residue in the X_3_ position), as reported in Table S4. Moreover, the four clusters and the three outliers differ for the side chain conformation of the coordinating residues. The analysis of the Cys and His χ_1_ angles allowed the main differences to be delineated among the clusters and to further group them into three subsets. In particular, the Cys and His χ_1_ angles adopts the following conformations, respectively: (i) *g*+, *g*− in the first subset, including clusters 1 and 3 (Cys χ_1_ = 72°± 5° and His χ_1_ = −72° ± 10°);( ii) *trans*, *g-* in the second subset, comprising clusters 2 and 4 and outliers 6 and 7 (Cys χ_1_ = 179° ± 6° and His χ_1_ = −58° ± 8°); (iii) *trans*, *g*+ in the last, represented by outlier 5 (Cys χ_1_ = −175° and His χ_1_ = 93°). In this perspective, clusters 3 and 4 can be considered as special cases of clusters 1 and 2, respectively. In these clusters, Cys residue adopts pre-Pro conformation and this finding is supported by the high occurrence of Pro residue at X_1_ position.

Concerning zinc coordination, the histidine residue utilizes its N^δ1^ atom to bind the zinc ion in all clusters. The binding site is characterized by S^γ^-Zn and N^δ^-Zn distances of (2.34 ± 0.07) Å and (2.1 ± 0.1) Å, respectively, in agreement with the bond lengths reported in previous surveys of zinc coordinating proteins [69,70].

In order to gain information about the homodimer topology, the database was further analyzed, searching for the propensity in symmetric dimer formation among the clusters. Unfortunately, no Cys_2_His_2_-containing dimer was found among the analyzed structures, and only four entries, belonging to clusters 2, 4 and outlier 5 (Table 5), displayed almost symmetrical Cys_3_His dimers, in which symmetry-related coordinating residues adopt the same χ_1_ torsion angles.

Two considerations originate from this analysis: (i) in all dimers, also comprising nonsymmetrical ones, the configuration around the metal ion is Λ; (ii) pseudo-symmetrical dimers were observed only when the χ_1_ torsion angles of Cys and His belongs to the second (*trans*; *g*−) or the third (*trans*; *g*+) subset. The interpretation of the structural determinants, responsible of the observed behavior in natural dimers, is premature at this stage; it will be subject of further study once more data from a complete database, comprising all the possible dimer combinations and metal ions, will be available.

### 2.7. Dimer Selection and Model Validation

**Figure 9:**
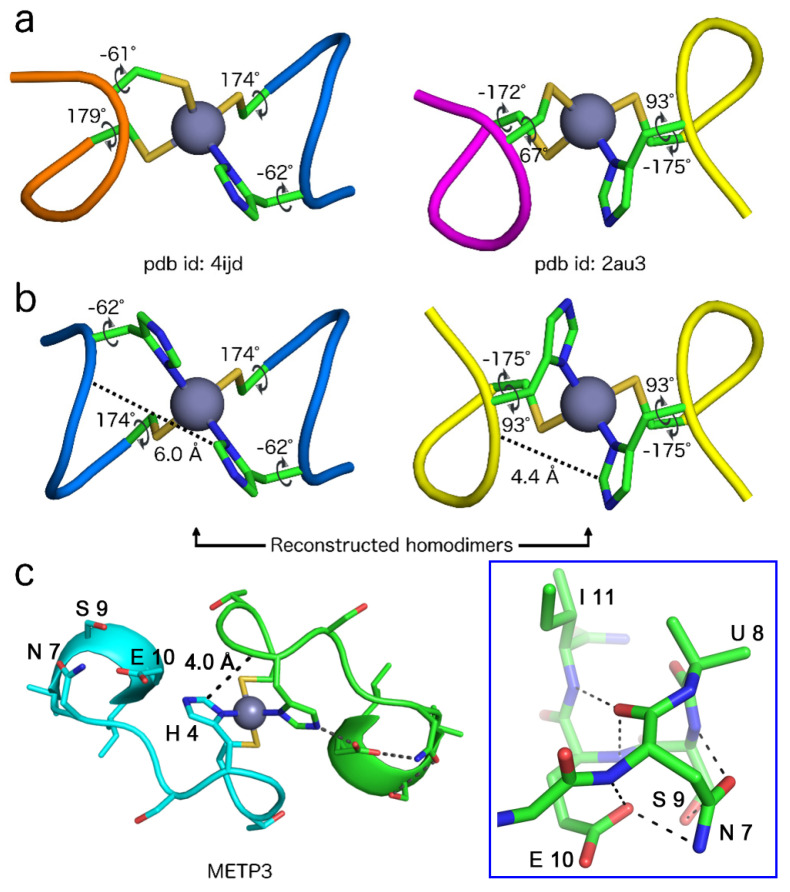
(**a**) Almost symmetrical Cys_3_His dimers, from 4ijd (left) and 2au3 (right), in which symmetry-related coordinating residues adopt the same χ_1_ torsion angles. (**b**) Possible homodimeric models, reconstructed from the C-X_1_-X_2_-H-X_3_ motifs, extracted from 4ijd (left) and 2au3 (right). (**c**) Final molecular model of Zn(II)-METP3 complex, with the observed hydrogen bond network, comprising Asn7-Aib8-Ser9-Glu10-Ile11, highlighted in the blue box. In all panels, the metal ions are reported as spheres and side chains as sticks.

The information deduced from the structural database analysis was used to select and construct a suitable dimeric structural model for the Zn(II)-METP3 complex, which may account for the NMR data. To this end, the crystallographic coordinates of the C-X_1_-X_2_-H-X_3_ motifs, extracted from 4ijd (belonging to cluster 2) and 2au3 (outlier 5) proteins, were used (Figure 9a). Applying the appropriate C_2_ symmetry operation, two possible dimeric structures, housing the Cys_2_His_2_ binding motif, were built (Figure 9b). 

Both the resulting dimers possess a Λ configuration around the metal, as expected for Zn(II)-METP3 complex, but only the dimer generated from 2au3 displays His H^ε1^ proton from one chain and His amide proton from the other at a sufficient distance to give rise to a dipolar contact (4.4 Å in 2au3 vs. 6.0 Å in 4ijd). Furthermore, in both dimers, the intra-residue H^ε1^/HN distance is 6 Å. Analysis of the reconstructed homodimer topologies suggest that the idealized 2au3-based symmetrical dimer represents a suitable model for NMR structure resolution. Indeed, in this model, the observed NOE cross-peak found in Zn(II)-METP3 complex can be unambiguously assigned as an inter-chain contact.

Taking into account all the NMR data and the idealized 2au3-based symmetrical dimer, an initial model of Zn(II)-METP3 complex was constructed. This model was then subjected to restrained energy minimization (REM). The backbone conformation of Zn(II)-METP3 complex after energy minimization is reported in Table S5 and the final minimized structure is reported in Figure 9c.

**Figure 10:**
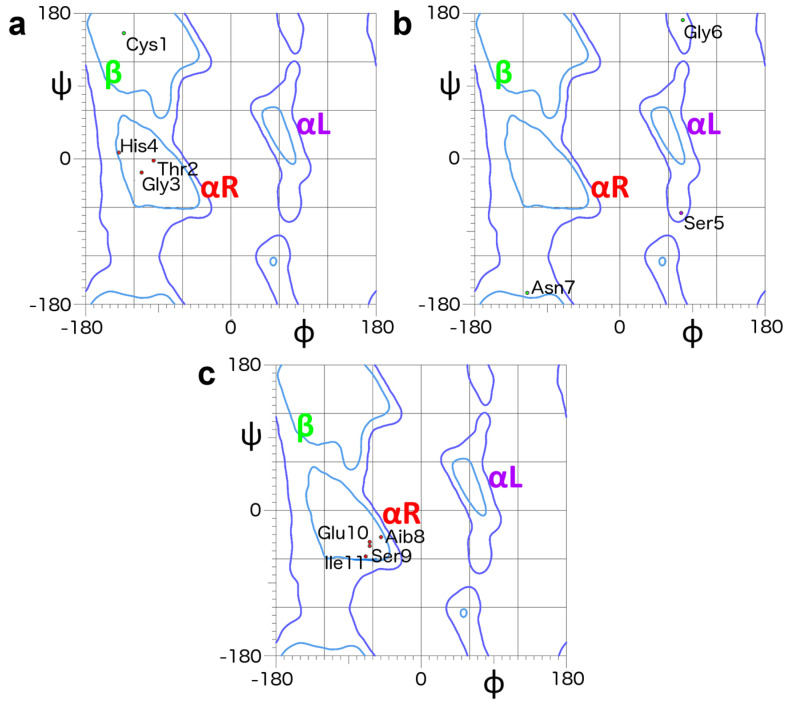
Ramachandran plots of the refined METP3 model. (**a**) Region from Cys^1^ to His^4^ encompassing a type Iα_RS_ turn. (**b**) Ser^5^ to Asn^7^ in the extended region. (**c**) Terminal helical region from Aib^8^ to Ile^11^. Color-coded labels define the most representative areas of the Ramachandran plot: right-handed α-helix/red, ß-strand/green, left-handed α-helix/violet. ϕ, ψ values of chain A are reported. Molprobity [71] has been used to generate the plot (http://molprobity.biochem.duke.edu/, accessed on 24 August 2021).

The molecule is characterized by three regions with different complexity and structural organization. Figure 10 illustrates the distribution of the ϕ_i_, ψ_i_ angles in Zn(II)-METP3. 

In the first region, corresponding to the N-terminal sequence (Ac^0^-His^4^), the Thr^2^-Gly^3^-His^4^ segment participates to an α-turn (Figure 10a). The values of dihedral angles for the residues Thr^2^, Gly^3^ and His^4^ (ϕ_2_, ψ_2_ = −74°, −21°, ϕ_3_, ψ_3_ = −86°, −8°, ϕ_4_, ψ_4_ = −157°, 6° chain A and ϕ_2_, ψ_2_ = −96°, −3°, ϕ_3_, ψ_3_ = −112°, −18°, ϕ_4_, ψ_4_ = −140°, −8° chain B), are very close to the values expected for a type Iα_RS_-turn [67].

The second region comprising the segment Ser^5^-Gly^6^, is characterized by dihedral angles typical of an extended conformation (Figure 10b). The values of dihedral angles for the residues belonging to this peptide segment are: ϕ_5_, ψ_5_ = 75°, -75°; ϕ_6_, ψ_6_ = 81°, 179° for chain A and ϕ_5_, ψ_5_ = 76°, −68°; ϕ_6_, ψ_6_ = 78°, 172° for chain B. However, the resulting arrangement of this regions could not be unambiguous, due to the low density of restraints.

The third region, corresponding to the C-terminal segment (Aib^8^-Ile^11^), is characterized by high structural organization and can be described as a C-capping motif (β-helix) [72], which caps an incipient helix (Aib^8^-Ile^11^) (Figure 9c blue box and Figure 10c). This structure is stabilized by an intramolecular hydrogen bond between the CO of Asn^7^ and the NH of Glu^10^, as well as the i, I + 4 hydrogen bond between the CO of Asn^7^ and the NH of Ile^11^. Further stabilization is given by a strong hydrogen bond interaction involving the side chain carbonyl of Asn^7^ and the amide of Ser^9^ (Figure 9c). The Glu^10^-His^4^ hydrogen bond, which was intended as an intermolecular interaction in the designed model, is retained as intramolecular in the refined model derived from the cluster analysis (Figure 9c). The full hydrogen bond network is reported in Table S6.

The overall fold of Zn(II)-METP3 is slightly looser with respect to the designed model, and the monomer poorly interacts with the symmetric counterpart. This finding is in agreement with the lack of strong intermolecular cross-peaks in the NOESY spectrum. Nevertheless, the single inter-chain NOE connectivity involving the ε^1^ and amide protons of His^4(4’)^ would be satisfied in the current refined model, where a distance of 4.0 Å is found between these two protons (Figure 9c). As a final remark, the overall looseness of the final model comes with no surprise. The TFE has a well-documented effect on protein folding when mixed with water, generally driving the increase in secondary structure content. However, its low dielectric constant (8.55) undoubtedly affects the entropically driven hydrophobic collapse of the dimer when used as the sole solvent. In this respect, the role exerted by the metal coordination is crucial in the peptide dimerization, as intended in the design. A more compact structure is therefore expected in water, and future studies will address this aspect.

## 3. Discussion

The aim of this work was to assess whether a short peptide sequence could be used as scaffold for developing metal binding sites capable of binding different metal ions with identical coordination geometries. This system could represent a simple and versatile platform for studying metal-coordination properties, evaluating the physical properties of the bound ions and the selectivity of the metals toward different coordination environments. 

METP3 was obtained by grafting a Cys_2_His_2_ metal binding site into the previously developed METP model, a small peptide containing a tetrahedral Cys_4_ coordination site. Similarly to its precursor, METP3 is a homodimer consisting of two undecapeptide chains, self-assembling in the presence of divalent metal ions. Three substitutions (Lys^3^Ala/Gly^5^Ser/Ala^6^Gly) proved necessary to complement the changes in the first coordination sphere, resulting in a small, dimeric system, able to host a tetrahedral Cys_2_His_2_ metal binding site. Indeed, the spectroscopic characterization demonstrated that METP3 binds transition metal ions with the predicted 2:1 peptide/metal stoichiometry and the desired tetrahedral geometry. In particular, the UV-visible spectral features of the Co(II)-METP3 complex are distinctive of a tetrahedral Cys_2_His_2_ metal binding environment [56,73] (Figure 2 and Table 1). The *K*_D_ values (Table 2) for METP3 toward different metal ions further confirm a tetrahedral arrangement of the ligands around the metal site [57,58,74,75]. Indeed, Co(II) ions experience a loss of ligand field stabilization energy (LFSE) in the transition from the octahedral hexa-aquo complex Co(H_2_O)_6_^2+^ to the tetrahedral Co(II)-peptide complex, whereas *d*^10^ ions (as Zn(II) and Cd(II)) have no LSFE for any coordination geometry [59]. Zn(II) is more efficient than Co(II) in preventing cysteine oxidation, further evidencing a tight and specific Zn(II)-peptide binding.

Conformational analysis by CD and NMR spectroscopies indicates that peptide fold is driven by metal ion coordination. In this respect, METP3 behaves as a minimal model of Zn-finger proteins. The observed CD spectral pattern of Zn(II)-METP3 complex can be attributed to a turned conformation such as α-helical turns, 3_10_ helical turns, or type I (III) β-turns, similarly to previous findings for the parent Zn(II)-METP complex [48]. 

The presence of turned structures was further confirmed by NMR studies of the Zn(II) complex. Even though high-resolution structural information could not be obtained solely from NMR data, a considerable help in outlining the dimer topology came from the analysis of zinc-containing protein structures in the PDB. The examined database was limited to high-resolution crystal structures of zinc-proteins with a Cys_x_His_y_ (x + y = 4; x, y ≠ 0) coordination environment in pseudo-symmetric dimers, where x and y refer to the number of Cys and His, respectively, located in the first coordination sphere. This analysis was not only helpful in solving the Zn(II)-METP3 structure, but also provided useful information on the molecular architecture of the CXXHX motif, both in terms of peptide conformation and zinc coordination geometry.

The CXXHX motif has been previously identified in several studies [76,77,78], as implicated in the formation of DNA binding domains (zinc fingers), or protein dimerization domains, although it has not been systematically studied in terms of its conformational preferences. Herein, by a search in the PDB database, we demonstrate that when Cys and His residues are both coordinated to zinc ion, the CXXHX motif predominantly adopts an α-turn conformation (type Iα_RS_). The selected Zn-sites were grouped into four clusters and three outlier structures (Figure 7), that mainly differ from each other for the side chain conformation of the coordinating residues. The position-dependent occurrence and propensity, at the X_1_, X_2_ and X_3_ positions, for each amino acid in the C-X_1_-X_2_-H-X_3_ α-turn motif, were also examined (Table 3). Gly is not allowed at the X1 and X2 positions, because they correspond to the i+1 position of type III and I β-turns composing the α-turn [79,80,81]. In contrast, Pro displays the highest propensity at the X_1_ position. Furthermore, the X_3_ position is optimal for C-cap interaction with the backbone carbonyl group of the Cys residue. These findings may provide useful information to guide future designs. Analysis of the structural database also provided insights into zinc coordination. All structures within our dataset exhibited a tetrahedral coordination around the metal ion, with the His binding through the N^δ^ atom. The preference of N^δ^ over N^ε^ histidine coordination is highly frequent among Zn-fingers, but the functional and structural significance of these two diverse binding modes remains not fully clarified [82,83].

Overall, the database analysis revealed that: (i) all the dimeric structures have a Λ configuration around the metal ion; (ii) no symmetric Cys_2_His_2_, but only Cys_3_His sites are found in pseudo-symmetrical dimers (C_2_-symmetry-related α-turns both showing the same χ_1_ conformation of coordinating residues); (iii) in all the symmetrical dimers, the Cys χ_1_ is *trans* conformation, while either a *g^+^* or *g^−^* conformation is adopted by the His χ_1_.

Interestingly, this analysis highlights that different χ_1_ angles adopted by the His residue (χ_1_ = 60.0° and −60.0°) may be responsible for different interactions involving His ε1 proton, and only one side chain rotamer justifies the observed inter-monomer NOE contact found in the NMR data. Based on these results, we generated a Zn(II)-MEPT3 symmetrical homodimer, consistent with the NMR data, using the 2au3_A protein motif as template. The minimized model is characterized by three regions with different complexity and structural organization (Figure 10), and it deviates from the backbone conformation of the original designed model only in the His^4^-Ser^6^ segment. 

In conclusion, the use of a combined approach of experimental data and statistical methods allowed to define the structural model of METP3, an artificial metalloprotein housing a Cys_2_His_2_ zinc binding site, unprecedented in nature.

## 4. Materials and Methods 

All 9-fluorenylmethoxycarbonyl (Fmoc) protected amino acids, Rink Amide MBHA resin and coupling reagents: N-hydroxybenzotriazole (HOBt) and benzotriazole-1-oxy-tris-pyrrolidino-phosphonium hexafluorophosphate (PyBOP) were purchased from NovaBiochem (EMD Biosciences, La Jolla, CA). All solvents used in the peptide synthesis and purification were anhydrous and High-Performance Liquid Chromatography (HPLC) grade, respectively, and were supplied by Romil. Piperidine. 3-(N-morpholino) propanesulfonic acid (MOPS), dimethylsulfoxide (DMSO) and scavengers (ethanedithiol, triisopropylsilane) were obtained from Sigma-Aldrich. N,N-Diisopropylethylamine (DIEA), trifluoroacetic acid (TFA) were supplied from Applied Biosystems. N,N-dimethylformamide (DMF), dichloromethane (DCM), pyridine, ethanol, methanol and 1-methyl-2-pyrrolidone (NMP) were supplied by Romil.

### 4.1. Instrumentations

Peptide synthesis was performed on an ABI 433 automatic peptide synthesizer using Fmoc solid phase peptide synthesis (SPPS). HPLC analysis were performed with a Shimadzu LC-10ADvp equipped with an SPDM10Avp diode-array detector. 

Mass spectra were acquired on a MALDI-TOF Voyager PerSeptive BioSystem.

UV-vis spectra were recorded on a Cary Varian 50 Spectrophotometer, equipped with a thermostated cell compartment (Varian, Palo Alto, CA, USA);CD analysis were performed using a J-815 spectropolarimeter equipped with a thermostated cell holder (JASCO, Easton, MD, USA). 

NMR experiments were acquired at 298 K on a Varian Inova spectrometer, operating at 500 MHz.

### 4.2. Peptide Synthesis and Purification

The undecapeptide Ac-Cys-Thr-Gly-His-Ser-Gly-Asn-Aib-Ser-Glu-Ile-NH_2_ was synthesized by standard Fmoc/tert-butyl (tBu) strategy, on a Rink Amide MBHA resin (0.65 mmol/g resin substitution). The synthetic procedure can be summarized as follows: The α-Fmoc group, was removed at the beginning of every cycle with a 20% piperidine solution in NMP. After deprotection, the resin was washed with NMP to remove the piperidine. The peptide resin was then ready for coupling; single coupling was conducted for each amino acid. Acylation reactions were carried out using a 0.5 M HOBT solution in DMF. In the coupling step, the activated Fmoc-amino acid reacts with the amino-terminal group of the growing peptide chain to form a peptide bond. Capping reaction was performed after each coupling step, using Ac_2_O/HOBt/DIEA solution in NMP. Deprotection, coupling and capping steps were repeated with each subsequent amino acid, until the chain assembly was completed. When the coupling was complete, the resin was washed with NMP. Peptides N-terminal amino groups were acetylated with Ac_2_O/HOBt/DIEA solution in NMP. Cleavage from the resin and sidechain deprotection was achieved with ethanedithiol/H_2_O/triisopropylsilane/TFA 0.25:0.25:0.1:9.5 (*v*/*v*/*v*/*v*) for 2 h. Extraction of the scavengers and precipitation of the crude product was achieved by addition of cold diethyl ether. The peptide was reduced by 3 h incubation in aqueous solution at pH 8, with 10-fold excess DTT. Purification by preparative RP-HPLC gave the desired pure product, as assayed by analytical HPLC (Vydac C8 column, using a gradient of acetonitrile in 0.1% aqueous TFA, 50% to 90% over 20 min, at a flow rate of 1.0 mL/min) and matrix-assisted laser desorption ionization time-of-flight spectrometry [molecular weight: calculated 1130.4 atomic mass units (amu); obs 1131 ± 1 amu] (Figures S1 and S2).

The inter-molecularly oxidized peptide was prepared by dissolving the reduced peptide (1 mM peptide concentration) in a MOPS/DMSO (90:10 *v*/*v*) solution at pH 6.5. The formation of the oxidized peptide was confirmed by RP-HPLC analysis (Figure S5).

### 4.3. Metal-Binding Experiments

All manipulations were performed under strictly anaerobic conditions. Both peptide and metal stock solutions were freshly prepared. 

The Co(II) and Zn(II) complexes were prepared by addition of CoCl_2_ or ZnCl_2_, in 10% molar excess, to an aqueous solution of the reduced METP3 monomer at pH ≈ 4 (1 mM peptide concentration); the pH was then raised to 8.2 by addition of TRIS buffer (10 mM final buffer concentration).

The pH dependence of the Co(II)-METP3 complex formation was determined by titrating an aqueous solution of the reduced peptide at 1.0 mM concentration, and of CoCl_2_ in 10% molar excess, with phosphate buffer, for the pH range 3.7–6.9 and with TRIS buffer for the pH range 7.2–9.2.

The binding affinity of Co(II) to METP3 peptide was measured by spectrophotometric titration of CoCl_2_ into the reduced peptide at 8.0 × 10^−4^ M concentration (10 mM Tris buffer, pH 8.2), under anaerobic conditions. The observed absorbance intensities were fit (KALEIDAGRAPH, Synergy Software (Reading, PA, USA); see Supplementary Material) to a binding isotherm [59,84] to estimate the dissociation constant *K*_D_ Co and the stoichiometry of the interaction n, according to the reaction:

1/nCoP_n_ = P + 1/nCo *K*_D_^Co^ = [Co]_f_^1/n^[P]_f_ / [CoP_n_]^1/n^ (1)

where [Co]_f_, [P]_f_, [CoP_n_] are the concentrations of free cobalt, free peptide (in monomeric units) and the complex, respectively.

The affinity of Zn(II) was estimated by competition experiments on the Co(II) complex. Aliquots of ZnSO_4_ aqueous solution (1.9 × 10^−2^ M) were titrated into a solution containing the reduced peptide at 1.0 × 10^−3^ M concentration, and 3.5-fold molar excess of Co(II) (10 mM Tris buffer, pH 8.2), under anaerobic conditions. The bleaching of the absorption spectrum was monitored.

The relative affinity of the interaction was obtained by fitting the data to the equilibrium [59]:

CoP_2_ + Zn^2+^·6H_2_O = ZnP_2_ + Co^2+^·6H_2_O *K*_ex_ = [ZnP_2_][Co]_f_/[CoP_2_][Zn]_f_ (2)

where [ZnP_2_], [CoP_2_], [Co]_f_, [Zn]_f_, are the concentrations of Zn(II) complex, Co(II) complex, free cobalt and free zinc ions, respectively. *K*_D_^Zn^ was determined from *K*_D_^Co^ and *K*_ex_: *K*_D_^Zn^ = *K*_D_^Co^/(*K*_ex_)^1/2^.

A similar experiment was performed in order to determine the affinity of Cd(II). Aliquots of CdCl_2_ aqueous solution (1.9 × 10^−2^ M) were titrated into a solution containing the reduced peptide at 1.0 × 10^−3^ M concentration, and 3.5-fold molar excess of Co(II) (10 mM Tris buffer, pH 8.2), under anaerobic conditions. The bleaching of the absorption spectrum was monitored. Similarly, to the experiment with Zn(II), the relative affinity of the interaction was obtained by fitting the data to the equilibrium:

CoP_2_ + Cd^2+^·6H_2_O = CdP_2_ + Co^2+^·6H_2_O *K*_ex_ = [CdP_2_][Co]_f_ / [CoP_2_][Cd]_f_ (3)

where [CdP_2_], [CoP_2_], [Co]_f_, [Cd]_f_, are the concentrations of Cd(II) complex, Co(II) complex, free cobalt and free cadmium ions, respectively.

### 4.4. UV-Visible (UV-Vis) Spectroscopy

UV-vis spectra were recorded on a Cary Varian 50 Spectrophotometer, equipped with a thermostated cell compartment (Varian, Palo Alto, CA, USA) by using quartz cuvettes with a path length of 1.0 cm. Wavelength scans were performed at 25 °C from 200 to 800 nm, with a 600 nm·min^−1^ scan speed. Anaerobic spectra were recorded in rubber-sealed cuvettes under nitrogen.

### 4.5. Circular Dichroism (CD) Spectroscopy

Data were collected at 25 °C, from 260 to 190 nm at 0.2 nm intervals with a 20 nm min^−1^ scan speed, at 2 nm band width and at 16 s response, and a 2 s response from 250 to 800 nm. Cells of 0.01 cm and 1 cm path length were used for the far UV and near UV-vis regions, respectively. Peptide concentrations were in the range 0.20–1.2 mM. CD intensities in the far UV and near UV-visible regions are expressed as mean residue ellipticities (deg cm^2^ dmol^−1^ res^−1^) and total molar ellipticities, respectively.

### 4.6. NMR Spectroscopy

NMR sample was prepared directly into an NMR tube by addition of NaOD (D_2_O solution, 1.5 equivalents) to a CF_3_CD_2_OH (TFE) solution of reduced METP3 monomer (2.0 mM) and ZnSO_4_ (aqueous solution, 1.1 equivalents). 3-(trimethylsilyl) propionic-2,2,3,3-D4 acid sodium salt (TSP) was added as internal reference. The NMR tube was flushed with nitrogen and sealed. 

Two-dimensional (2D) experiments, such as total correlation spectroscopy, nuclear Overhauser effect spectroscopy (NOESY) and double quantum filtered correlation spectroscopy (DQFCOSY) were recorded by the phase-sensitive States-Haberkorn method [85]. Total correlation spectroscopy experiments were acquired with a 70 ms mixing time, whereas NOESY experiments were acquired with 100, 200, and 300 ms mixing times. 

According to Wüthrich [63], identification of amino acid spin systems was performed by comparison of TOCSY and DQFCOSY, while sequential assignment was obtained by the analysis of NOESY spectra. NOE intensities were evaluated by integration of cross-peaks in the 300 ms NOESY spectrum.

All two-dimensional data consisted of 4K data points in the direct dimension. There were 512 experiments recorded in the indirect dimension, using 64 scans per experiments. Raw data were multiplied in both dimensions using a Gaussian function. A polynomial base-line correction was applied in both dimensions.

Semi-quantitative information on inter-proton distances for structure calculation were obtained by analyzing the 300 ms NOESY spectrum that did not exhibit spin diffusion effects. ^3^JNH-αCH coupling constant values were obtained from 1D and 2D spectra, and the related ϕ dihedral angles were computed according to Karplus [64].

The proton chemical shifts, coupling constants and temperature coefficients are reported in Table S1. The NOESY experiments yielded about 80 NOE contacts in positive regime.

### 4.7. Analysis of the Protein Structure Database

Analysis of conformational preferences of the Cys-X_1_-X_2_-His-X_3_ sequence (X_1_, X_2_, X_3_ = any amino acid) was performed by generating a protein structure database from the Protein Data Bank (http://www.pdb.org, accessed on 14 September 2020), containing zinc-binding proteins sharing the following sequence pattern to prevent His-tag occurrence: XXXXXXXCXXHXXXXXXX.

The database started with approximately 350 protein structures solved by X-ray crystallography, at a resolution of 2.4 Å or better, and with a sequence identity cut-off up to 50%. In the case of proteins having multiple chains with the same sequence, only one chain was considered. 

As the first step of our database structural analysis, we found all the C-X_1_-X_2_-H-X_3_ motifs, in which both Cys and His residues are bound to zinc, and we restricted our analysis to those adopting an α-turn conformation. Thus, we selected, from the PDB, only the motifs showing Cα_i_-Cα_i+4_ distance ≤ 7 Å. We grouped together the Zn-sites, thus generating a structural database, which comprised 129 structures, 44 of which display a unique C-X_1_-X_2_-H-X_3_ sequence (Tables S2 and S3). 

All structures were manually inspected to assess zinc coordinating ligands, metal-to-ligand bond lengths, and coordination geometries (see Table S4). The GROMOS clustering algorithm [86], implemented in g_cluster, was used to determine cluster memberships with a 0.04 nm RMSD as calculated on an 18 atoms subset as showed in Figure S12. The structure with the greatest number of neighbors was used as the center of the cluster.

### 4.8. METP3 Model Refinement

The program package InsightII/Discover with the Covalent Valence Force Field (CVFF) [87,88], was used for energy minimization and RMD simulation. The designed monomer model was used as an initial model; it was examined for inconsistencies with the experimental NMR data (interproton contacts and ^3^J_NH-αCH_ coupling constants), and then subjected to RMD calculations, in vacuo at 300 K. Three classes of distance restraints were adopted according to the relative intensity of the integrated NOE signals: 1.8 ± 2.5 (strong), 1.8 ± 3.3 (medium), 2.7 ± 5.0 (weak). RMD calculations were run replacing the Zn^2+^ ion with Na^+^, due to the lack of an implemented force field for Zn^2+^ in the Discover package. The coordination geometry was simulated as follows: (a) the position of Na^+^ ion, Cys-S and His-N^δ1^ atoms were fixed; (b) distance restraints were introduced between the N^δ1^, S atoms and Na^+^ ion by using a force constant stronger than that used for the interproton distances accounting for the NOE effects. The distances were allowed to deviate in a range of ± 0.2 A. Reference distances were selected from the X-ray structures of Zn^2+^coordinating proteins.

A distance dependent dielectric constant was used through all computations. Leapfrog integration algorithm was adopted, with a time step of 1 fs. The protocol consisted of an equilibration phase of 50 ps and of a simulation phase of 360 ps. A structure was saved every 100 fs during the simulation for analysis. The final average structure was checked for consistency with all observable NOE. 

Final dimeric model was constructed as described in Section 2.7, under Accelrys Discovery Studio 3.0 environment. Briefly, 2au3_A CXXHX motif was used as template for Zn(II) complexation. A *C_2_* operation was performed to generate the symmetric tetrahedral site, and peptide sequence was mutated to the METP3 N-terminal sequence (from residue 1 to 5). Then, the C-terminal traits were grafted onto the freshly obtained dimer, by superimposing the average coordinates of the C-terminal turn (Gly_6_-Ile_11_) from RMD simulation. Only minimal manual manipulation was needed to properly link the N- and C-terminal structural units. Then, a NOE restrained minimization was performed under CHARMM27 forcefield parameterization, with a Newton–Raphson algorithm under an implicit Generalized Born solvent model [89]. Finally, an unrestrained minimization was performed and found only negligible deviation from the NOE-distance restrained model.

## Figures and Tables

**Table 1:** UV-visible spectral data of Co(II) complexes of METP3.

Compound	Wavelength [nm] (ε × 10^−3^ [M^−1^cm^−1^])				Coordination Site
METP3	308 (2.10)	564 (0.313)	624 (0.470)	649 (0.440)	Cys_2_His_2_
GGG	285 (3.4)	550 (0.220)	625 (0.500)	660 (0.430)	Cys_2_His_2_
CP-1	310 (2.50)	570 (0.300)		640 (0.900)	Cys_3_His
METP	353 (3.57)	624 (0.420)	685 (0.617)	735 (0.582)	Cys_4_

**Table 2:** *K*_D_ values for METP and METP3 peptides toward different metal ions.

Metal Ion	*K*_D_ (µM) METP ^a^	*K*_D_ (µM) METP3
Co(II)	(53.5 ± 2.8)	(85 ± 4)
Zn(II)	(2.7 ± 0.1)	(5.6 ± 0.2)
Cd(II)	/	(5.0 ± 1.0)

^a^ Data for METP are taken from [48].

**Table 3:** Occurrences (first line) and propensities ^a^ (second line) for the proteogenic amino acids from the non-redundant -C-X1-X2-H-X3- structural database.

Residue	X_1_	X_2_	X_3_
A	3	3	1
	0.74	0.74	0.25
C	0	0	0
	0	0	0
D	1	1	0
	0.41	0.41	0
E	6	6	8
	2.20	2.20	2.93
F	0	1	1
	0	0.58	0.58
G	0	0	7
	0	0	2.17
H	1	4	3
	1.03	4.13	3.10
I	1	3	0
	0.41	1.23	0
K	5	2	4
	2.32	0.93	1.86
L	2	0	2
	0.46	0	0.46
M	0	0	0
	0	0	0
N	2	3	2
	1.21	1.81	1.21
P	8	3	3
	3.69	1.38	1.38
Q	4	0	0
	2.41	0	0
R	6	3	1
	2.34	1.17	0.39
S	0	3	2
	0	1.02	0.68
T	3	3	3
	1.23	1.23	1.23
V	1	4	3
	0.33	1.31	0.98
W	1	1	0
	1.75	1.75	0
Y	0	4	4
	0	3.16	3.16

^a^ Propensities are calculated as reported in [66].

**Table 4:** Average dihedral angles of clusters expressed in degrees. The number of structures per cluster is shown in the brackets of the first column. Standard deviations are reported in parentheses.

Cluster	ϕ_i+1_	ψ_i+1_	ϕ_i+2_	ψ_i+2_	ϕ_i+3_	ψ_i+3_	χ_1_ Cys	χ_1_ His
1 (86)	−59 (5)	−37 (8)	−65 (7)	−47 (12)	−79 (18)	−21 (19)	72 (4)	−75 (8)
2 (16)	−63 (6)	−26 (11)	−91 (13)	−34 (12)	−116 (12)	10 (32)	177 (6)	−61 (6)
3 (13)	−63 (3)	−31 (10)	−79 (3)	−41 (3)	−116 (7)	−6 (2)	67 (3)	−53 (4)
4 (11)	−62 (8)	−40 (11)	−83 (9)	−33 (13)	−119 (12)	78 (9)	−178 (3)	−51 (6)
5 (1)	−86	−3	−110	−24	−135	158	−175	93
6 (1)	−58	−48	−59	−54	−63	−45	−177	−81
7 (1)	−121	−37	−57	−30	−115	18	178	−50

**Table 5:** Pseudo-symmetric Cys_3_His dimers observed in the database.

Entry	#Clust.	Sequence	χ_1_ Cys ^a^	χ_1_ His ^a^	Sequence	χ_1_ Cys ^a^	χ_1_ Cys ^a^
2v0c_A	4	^176^C-W-R-H-E^180^	174	−59	^159^C-P-K-C-Q^163^	177	−65
4ijd_A	2	^216^C-A-A-H-G^220^	174	−62	^205^C-E-M-C-Q^209^	179	−61
4ijd_B	2	^216^C-A-A-H-G^220^	−176	−56	^205^C-E-M-C-Q^209^	177	−57
2au3_A	5	^35^C-P-F-H-P^39^	−175	93	^56^C-F-G-C-G^60^	−172	67

^a^ χ_1_ dihedral angles are expressed in degrees.

## Data Availability

The data presented in this study are available in Tables S1–S7 of the Supplementary Information. HPLC, UV-Vis, CD, and NMR data are available upon request. The examined protein structures are available in the Protein Data Bank (https://www.rcsb.org, accessed on 15 September 2020).

## Supplementary Materials

The following are available online, Figure S1: RP-HPLC chromatogram at λ = 210 nm of pure METP3, Figure S2: MALDI-TOF spectrum of pure METP3, Figure S3: Visible spectra of Co(II)-METP3 complex in the absence and in the presence of 10% excess of Zn(II), pH = 8.2, Figure S4: Spectrophotometric competition experiment of Co(II)-METP3 complex with Cd(II). The saturation fraction 2[CdP2]/[P]t is plotted against the concentration ratio [Cd]t/[P]t. Solid line shows the best fit of data points to equation (3), Figure S5: RP-HPLC chromatogram at λ = 210 nm of inter-molecularly oxidized METP3, Figure S6: RP-HPLC chromatograms at λ = 210 nm of METP3 metal complexes. (a) Zn(II)-METP3 complex 4 days after the preparation; (b) Co(II)-METP3 complex 2 h after the preparation, Figure S7: Monomeric peptide chain of Zn(II)-METP3 complex: backbone atom superposition of an ensemble of 30 representative NMR conformers along the RMD trajectory, Figure S8: Monomeric peptide chain of Zn(II)-METP3 complex: backbone atom superposition of the N-terminal (a) and C-terminal (b) segments in the ensemble of 30 representative NMR conformers along the RMD trajectory, Figure S9: NOESY contour plot of the amide proton region of Zn(II)METP3 complex (mixing time 300 ms), Figure S10: Pie chart showing the functions of proteins making up the C-X1-X2-H-X3 structural database, Figure S11: Web logo analysis of the C-X1-X2-H-X3 motifs for: (a) the full dataset; (b) cluster 1; (c) cluster 2; (d) cluster 3; (e) cluster 4, Figure S12: Atom set used to superimpose the structures, Table S1: Proton Chemical Shifts, 3J Coupling Constants and Temperature Coefficients of Zn(II)-METP3 in TFE, at 298 K, Table S2: Protein structures containing the C-X1-X2-H-X3 motif (X1, X2, X3= any amino acid). The corresponding sequences are indicated as single Letter Codes. Protein function is also indicated, Table S3: Non-redundant C-X1-X2-H-X3 motif and their classification (X1, X2, X3 = any amino acid), Table S4: Database entries: conformational and metal coordination parameters for each C-X1-X2-H-X3 motif (X1, X2, X3 = any amino acid), Table S5: Torsion angles of Zn(II)-METP3 complex as obtained from REM (first row Chain A, second row Chain B) calculations in vacuo at 300 K, Table S6: Intramolecular hydrogen bonds of Zn(II)-METP3 complex as obtained from REM calculations in vacuo at 300K.
