# Supplementary material for: Unravelling the Structure of the Tetrahedral Metal-Binding Site in METP3 through an Experimental and Computational Approach
Source: Molecules. 2021 Aug 28;26(17):5221. doi: 10.3390/molecules26175221 (PMC8434281; doi:10.3390/molecules26175221)
Supplement: Supplementary file 1 [file molecules-26-05221-s001.zip › molecules-1326066-supplementary.pdf]

Supplementary Information

Unravelling the structure of tetrahedral metal binding sites through an experimental and computational approach

Salvatore La Gatta [1,†], Linda Leone [1,†], Ornella Maglio [1,2], Maria De Fenza [1], Flavia Nastri [1], Vincenzo Pavone [1], Marco Chino [1,*] and Angela Lombardi [1,*]

[1] Department of Chemical Sciences, University of Napoli Federico II, Via Cintia, 80126 Napoli, Italy

[2] Istituto di Biostrutture e Bioimmagini (IBB), National Research Council (CNR), Via Mezzocannone 16, 80134 Napoli, Italy

* Corresponding authors
† These authors contributed equally to this work.

SUPPLEMENTARY FIGURES

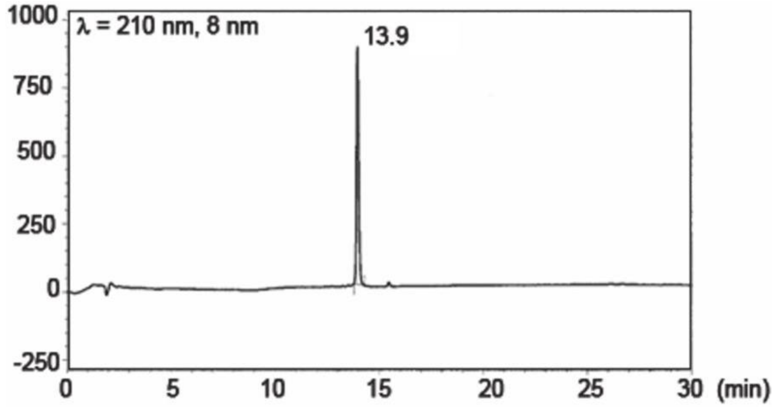

Figure S1. RP-HPLC chromatogram at λ = 210 nm of pure METP3.

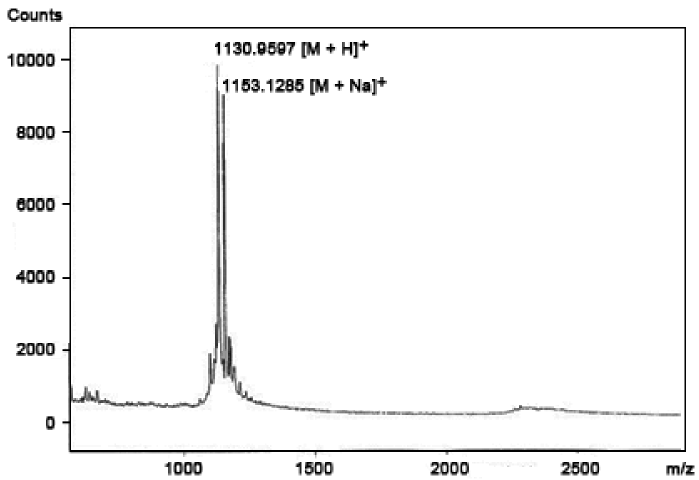

Figure S2. MALDI-TOF spectrum of pure METP3.

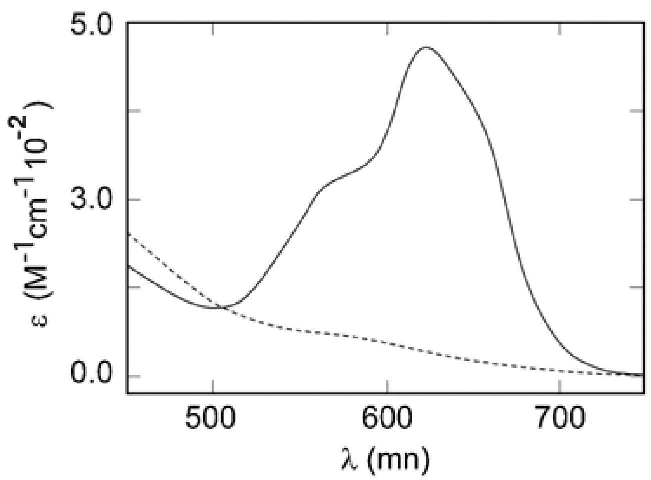

Figure S3. Visible spectra of Co(II)-METP3 complex in the absence (dotted line) and in the presence (solid line) of 10% excess of Zn(II), pH= 8.2.

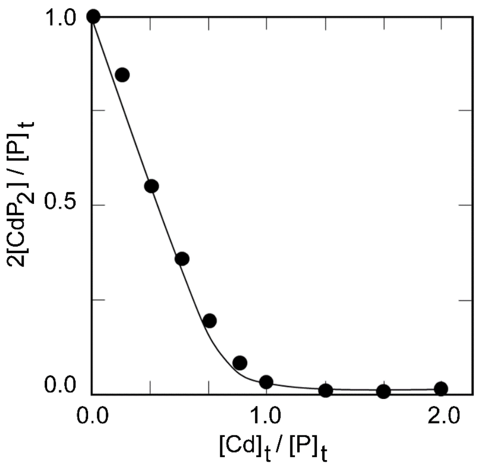

Figure S4. Spectrophotometric competition experiment of Co(II)-METP3 complex with Cd(II). The saturation fraction $2[CdP_2]/[P]t$ is plotted against the concentration ratio [Cd]t/[P]t. Solid line shows the best fit of data points to equation (3) reported in the main text.

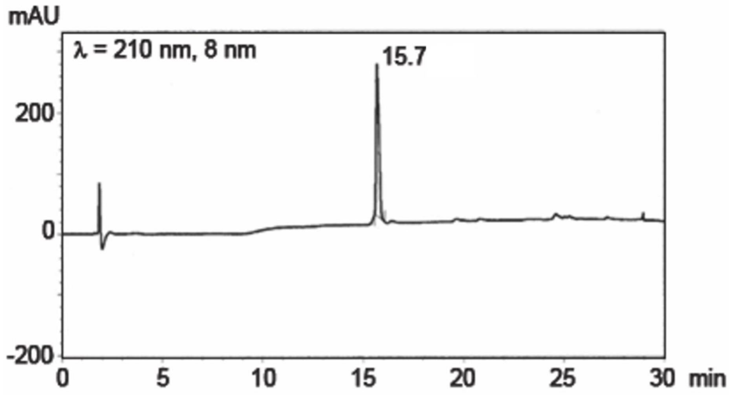

Figure S5. RP-HPLC chromatogram at λ = 210 nm of inter-molecularly oxidized METP3.

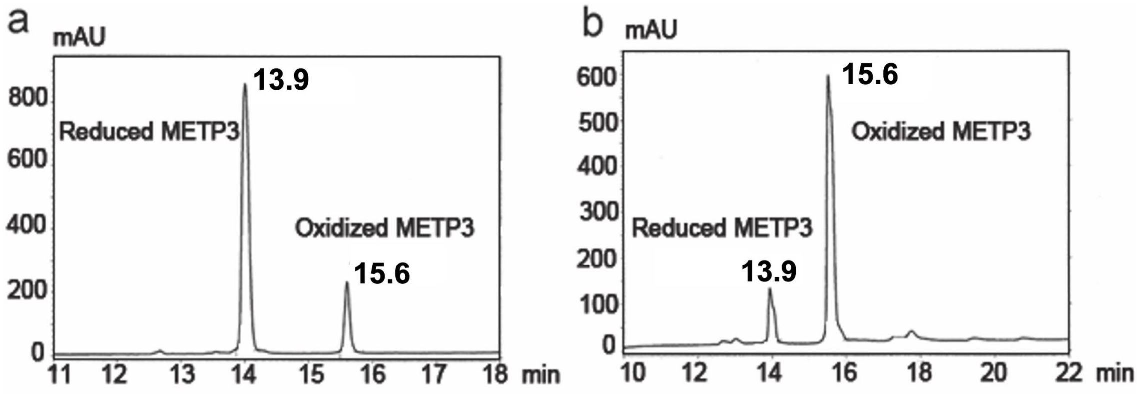

Figure S6. RP-HPLC chromatograms at λ = 210 nm of METP3 metal complexes. (**a**) Zn(II)-METP3 complex 4 days after the preparation; (**b**) Co(II)-METP3 complex 2 hours after the preparation.

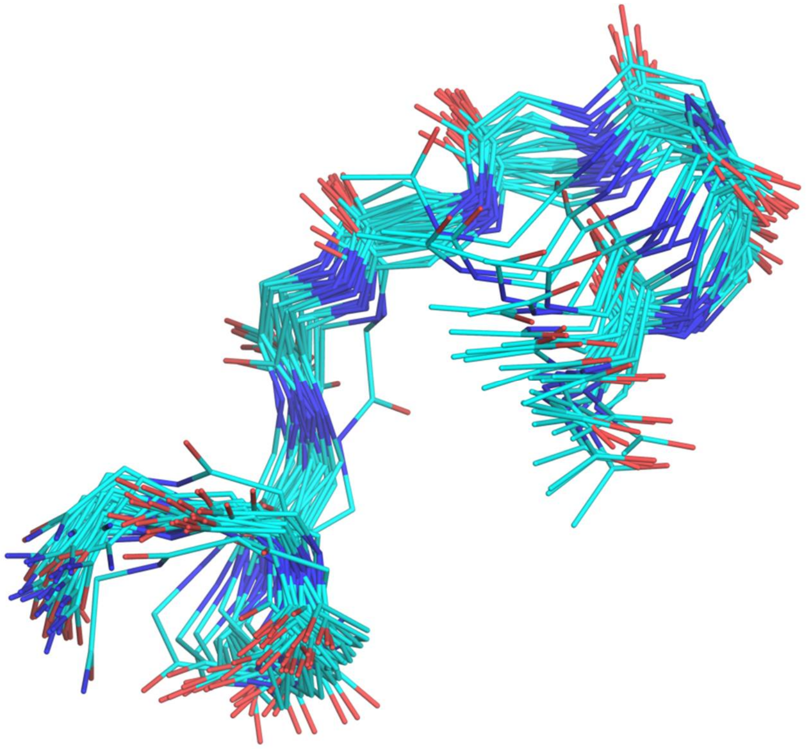

Figure S7. Monomeric peptide chain of Zn(II)-METP3 complex: backbone atom superposition of an ensemble of 30 representative NMR conformers along the RMD trajectory.

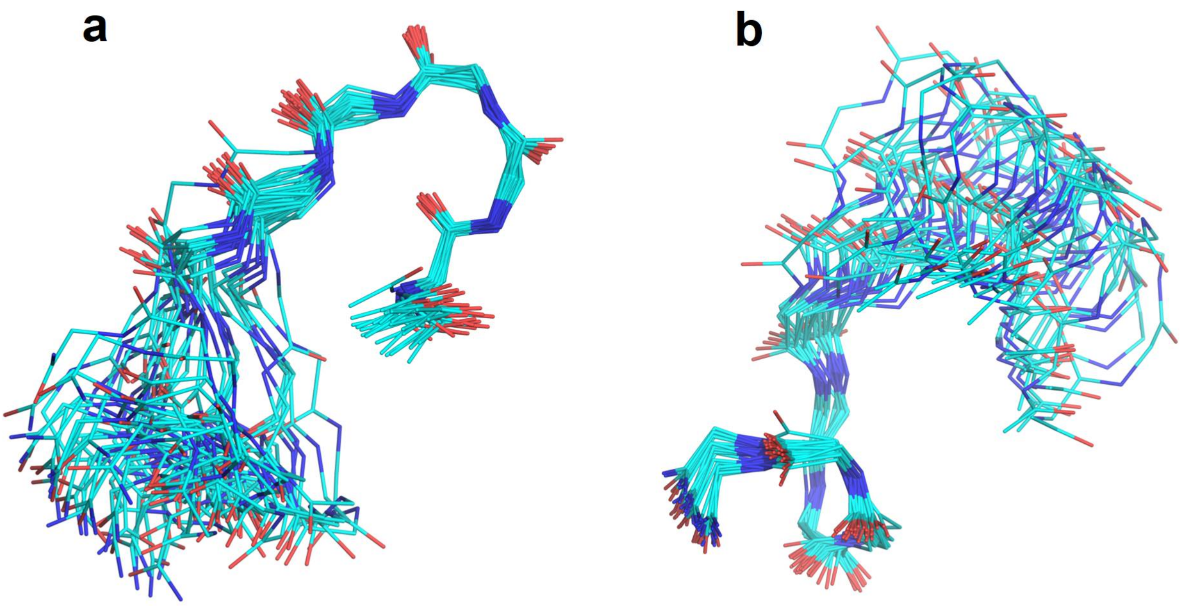

Figure S8. Monomeric peptide chain of Zn(II)-METP3 complex: backbone atom superposition of the N-terminal (**a**) and C-terminal (**b**) segments in the ensemble of 30 representative NMR conformers along the RMD trajectory.

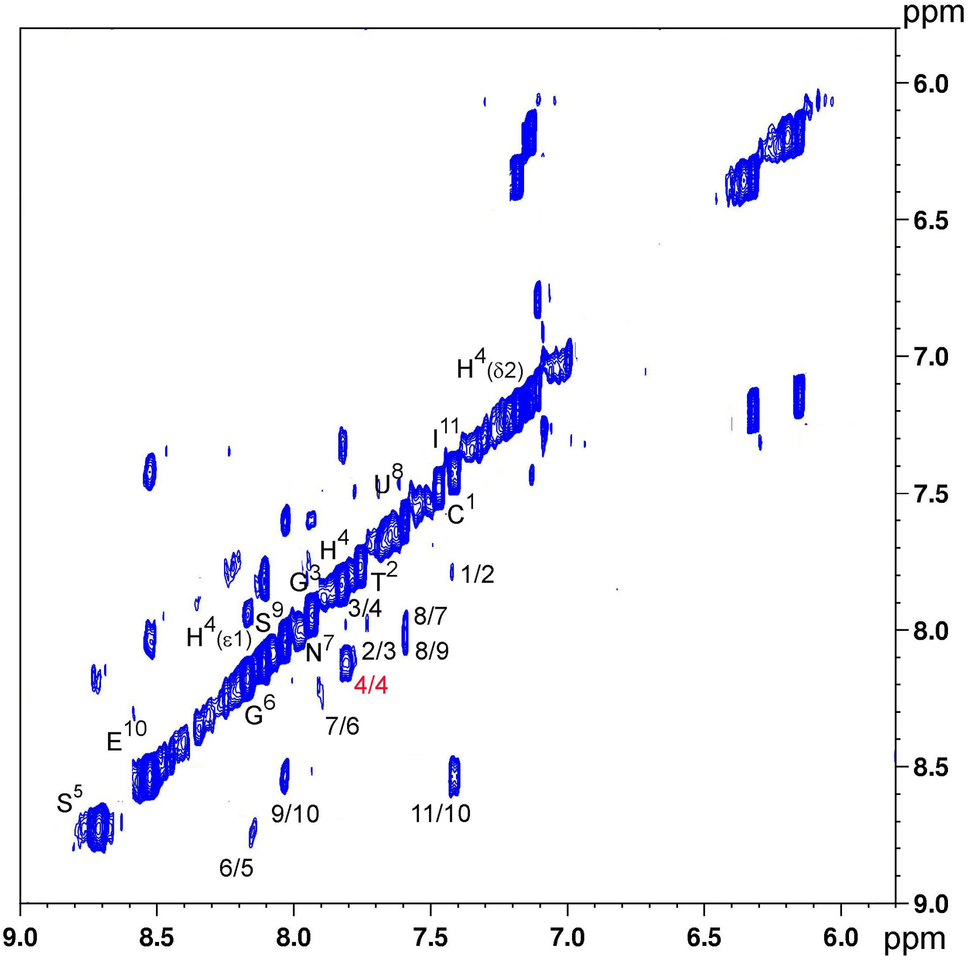

Figure S9. NOESY contour plot of the amide proton region of Zn(II)-METP3 complex (mixing time 300 ms). NH-NH connectivities are labeled. The intermonomer correlation involving the ε^1 and amide protons of His$^{4(4')}$ is labeled in red.

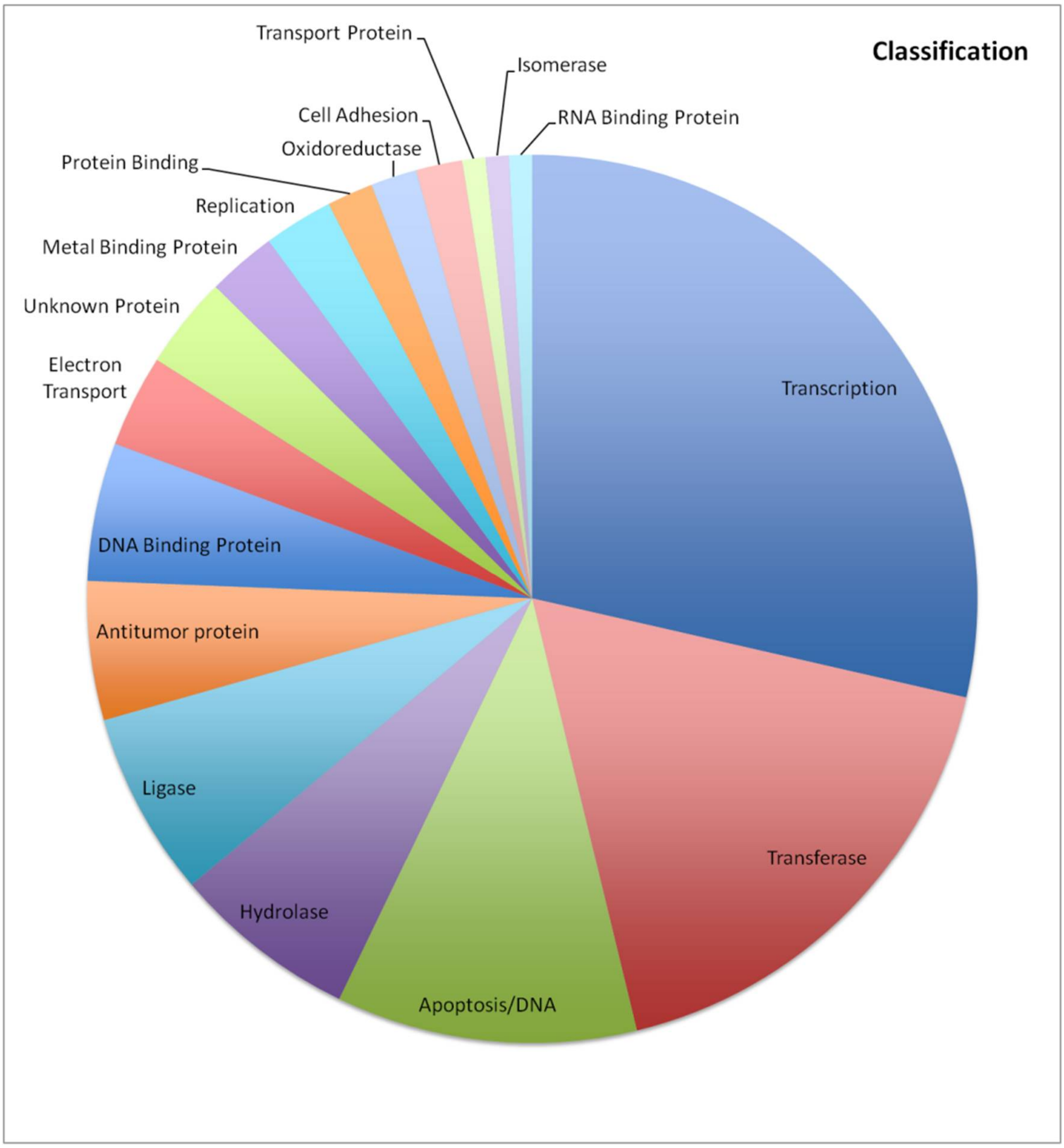

Figure S10. Pie chart showing the functions of proteins making up the C-X_1-X_2-H-X_3 structural database.

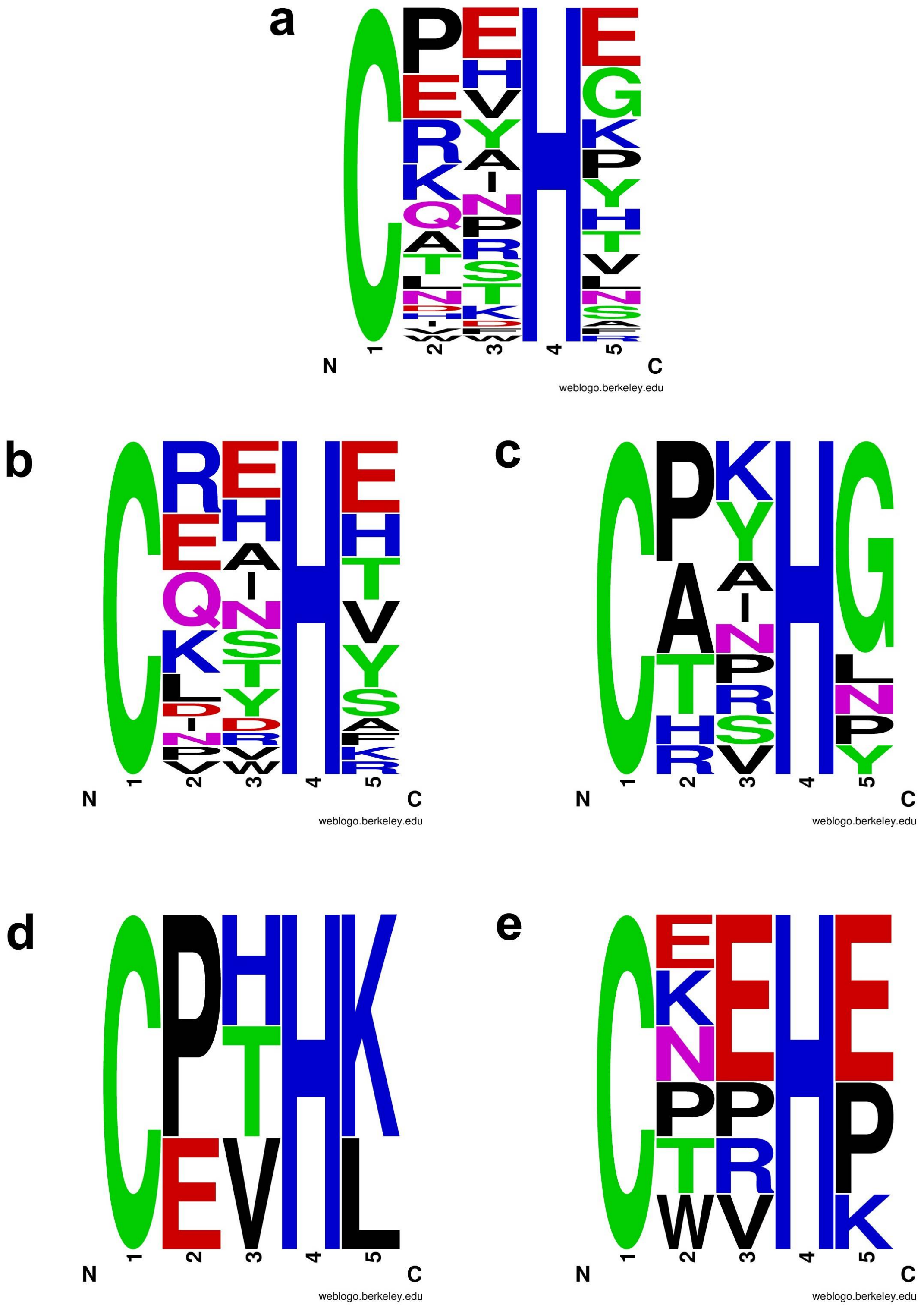

Figure S11. Web logo analysis of the C-X_1-X_2-H-X_3 motifs for: (**a**) the full dataset; (**b**) cluster 1; (**c**) Cluster 2; (**d**) Cluster 3; (**e**) Cluster 4.

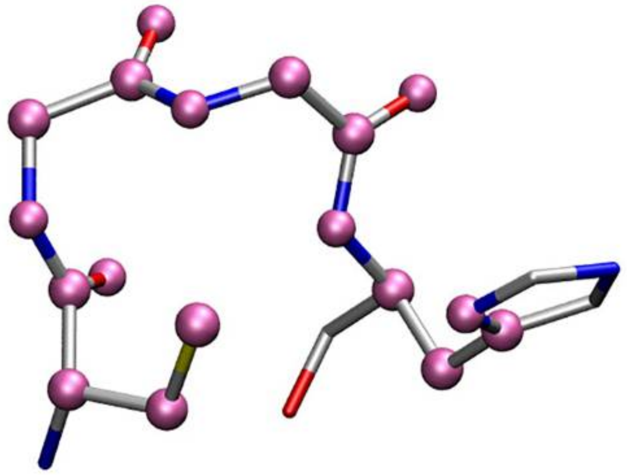

Figure S12. Atom set used to superimpose the structures. The atoms included in the data set are highlighted by pink spheres.

SUPPLEMENTARY TABLES

Table S1. Proton Chemical Shifts, ^{3}J Coupling Constants and Temperature Coefficients of Zn(II)-METP3 in TFE, at 298 K.[a]

Residue	NH	CαH	CβH	CγH	CδH	others	$^3J_{NH\text{-}\alpha CH}$ (Hz)	$-\Delta\delta_{NH}/\Delta T$ (ppb/K)
Acetyl						2.07		
Cys[1]	7.46	4.58	2.95-2.85				6.9	6.0
Thr[2]	7.75	4.33	4.33	1.25			6.6	4.3
Gly[3]	7.93	3.99-3.78					6.5	2.5
His[4]	7.82	4.78	3.32-3.10			8.10(ε1)-7.10(δ2)	7.0	7.1
Ser[5]	8.70	4.41	4.02-3.94				6.8	10.0
Gly[6]	8.16	4.03-3.90					8.2	4.3
Asn[7]	7.94	4.68	2.83			7.18-6.31(γNH_2)	7.7	4.2
Aib[8]	7.60		1.53^{proR}, 1.50^{proS}				-	4.1
Ser[9]	8.03	4.17	3.99-3.90				4.2	5.1
Glu[10]	8.52	4.20	2.16	2.45			7.8	5.3
Ile[11]	7.42	4.21	1.98	CγH_2 1.54-1.25<br>CγH_3 0.96	0.88		8.3	2.1
NH_2						7.13-6.15		3.8, 3.2

[a]Chemical shifts were referenced to the internal TSP.

Table S2. Protein structures containing the -C-X_1-X_2-H-X_3 motif (X_1, X_2, X_3= any amino acid). The corresponding sequences are indicated as single Letter Codes. Protein function is also indicated.

PDB code[a]	Macromolecular Name	Title	Classification	Resolution (Å)	-C-X_1-X_2-H-X_3-motif	Reference
1T4W_A	C.Elegans p53 tumor suppressor-like transcription factor	Structural Differences in the DNA Binding Domains of Human p53 and its C. elegans Ortholog Cep-1: Structure of C. elegans Cep-1	Transcription	2.10	-^{307}C-R-S-H-T^{311}-	Huyen Y. *et al.* Structure 2004, 12, 1237
1TSR_A	P53 tumor suppressor	P53 core domain in complex with DNA	Transcription	2.20	-^{176}C-P-H-H-E^{180}-	Cho Y. *et al.* Science 1994, 265, 346
1TSR_B	P53 tumor suppressor	P53 core domain in complex with DNA	Transcription	2.20	-^{176}C-P-H-H-E^{180}-	Cho Y. *et al.* Science 1994, 265, 346
1TSR_C	P53 tumor suppressor	P53 core domain in complex with DNA	Transcription	2.20	-^{176}C-P-H-H-E^{180}-	Cho Y. *et al.* Science 1994, 265, 346
1TUP_A	P53 tumor suppressor	Tumor suppressor P53 complex with DNA	Transcription	2.20	-^{176}C-P-H-H-E^{180}-	Cho Y. *et al.* Science 1994, 265, 346
1TUP_B	P53 tumor suppressor	Tumor suppressor P53 complex with DNA	Transcription	2.20	-^{176}C-P-H-H-E^{180}-	Cho Y. *et al.* Science 1994, 265, 346
1TUP_C	P53 tumor suppressor	Tumor suppressor P53 complex with DNA	Transcription	2.20	-^{176}C-P-H-H-E^{180}-	Cho Y. *et al.* Science 1994, 265, 346
1VFY_A	Phosphatidylinoditol-3-phosphate binding FYVE domain of protein VPS27	Phosphatidylinoditol-3-phosphate binding FYVE domain of VPS27P protein from *Saccharomyces Cerevisiae*	Transport Protein	1.15	-^{200}C-Q-E-H-S^{204}-	Misra S. *et al.* Cell 1999, 97, 657-666.
1WUR_A	GTP cyclohydrolase I	Structure of GTP cyclohydrolase I Complexed with 8-oxo-dGTP	Hydrolase	1.82	-^{108}C-E-H-H-L^{112}-	Tanaka Y. *et al.* J. Biochem. 2005, 138, 263
1WUR_B	GTP cyclohydrolase I	Structure of GTP cyclohydrolase I Complexed with 8-oxo-dGTP	Hydrolase	1.82	-^{108}C-E-H-H-L^{112}-	Tanaka Y. *et al.* J. Biochem. 2005, 138, 263
1WUR_C	GTP cyclohydrolase I	Structure of GTP cyclohydrolase I Complexed with 8-oxo-dGTP	Hydrolase	1.82	-^{108}C-E-H-H-L^{112}-	Tanaka Y. *et al.* J. Biochem. 2005, 138, 263

1WUR_D	GTP cyclohydrolase I	Structure of GTP cyclohydrolase I Complexed with 8-oxo-dGTP	Hydrolase	1.82	-^{108}C-E-H-H-L^{112}-	Tanaka Y. *et al.* J. Biochem. 2005, 138, 263
1WUR_E	GTP cyclohydrolase I	Structure of GTP cyclohydrolase I Complexed with 8-oxo-dGTP	Hydrolase	1.82	-^{108}C-E-H-H-L^{112}-	Tanaka Y. *et al.* J. Biochem. 2005, 138, 263
1YCS_A	P53 tumor suppressor	P53-53BP2 Complex	Transcription	2.20	-^{176}C-P-H-H-E^{180}-	Gorina S. *et al.* Science 1996, 274, 1001
2AC0_A	Cellular tumor antigen p53	Structural Basis of DNA Recognition by p53 Tetramers (complex I)	Apoptosis/DNA	1.80	-^{176}C-P-H-H-E^{180}-	Kitayner M. *et al.* Mol. Cell 2006, 22, 741
2AC0_B	Cellular tumor antigen p53	Structural Basis of DNA Recognition by p53 Tetramers (complex I)	Apoptosis/DNA	1.80	-^{176}C-P-H-H-E^{180}-	Kitayner M. *et al.* Mol. Cell 2006, 22, 741
2AC0_C	Cellular tumor antigen p53	Structural Basis of DNA Recognition by p53 Tetramers (complex I)	Apoptosis/DNA	1.80	-^{176}C-P-H-H-E^{180}-	Kitayner M. *et al.* Mol. Cell 2006, 22, 741
2AC0_D	Cellular tumor antigen p53	Structural Basis of DNA Recognition by p53 Tetramers (complex I)	Apoptosis/DNA	1.80	-^{176}C-P-H-H-E^{180}-	Kitayner M. *et al.* Mol. Cell 2006, 22, 741
2AHI_A	Cellular tumor antigen p53	Structural Basis of DNA Recognition by p53 Tetramers (complex III)	Apoptosis/DNA	1.85	-^{176}C-P-H-H-E^{180}-	Kitayner M. *et al.* Mol. Cell 2006, 22, 741
2AHI_B	Cellular tumor antigen p53	Structural Basis of DNA Recognition by p53 Tetramers (complex III)	Apoptosis/DNA	1.85	-^{176}C-P-H-H-E^{180}-	Kitayner M. *et al.* Mol. Cell 2006, 22, 741
2AHI_C	Cellular tumor antigen p53	Structural Basis of DNA Recognition by p53 Tetramers (complex III)	Apoptosis/DNA	1.85	-^{176}C-P-H-H-E^{180}-	Kitayner M. *et al.* Mol. Cell 2006, 22, 741
2AHI_D	Cellular tumor antigen p53	Structural Basis of DNA Recognition by p53 Tetramers (complex III)	Apoptosis/DNA	1.85	-^{176}C-P-H-H-E^{180}-	Kitayner M. *et al.* Mol. Cell 2006, 22, 741
2ATA_A	Cellular tumor antigen p53	Structural Basis of DNA Recognition by p53 Tetramers (complex II)	Apoptosis/DNA	2.20	-^{176}C-P-H-H-E^{180}-	Kitayner M. *et al.* Mol. Cell 2006, 22, 741
2ATA_B	Cellular tumor antigen p53	Structural Basis of DNA Recognition by p53 Tetramers (complex II)	Apoptosis/DNA	2.20	-^{176}C-P-H-H-E^{180}-	Kitayner M. *et al.* Mol. Cell 2006, 22, 741
2ATA_C	Cellular tumor antigen p53	Structural Basis of DNA Recognition by p53 Tetramers (complex II)	Apoptosis/DNA	2.20	-^{176}C-P-H-H-E^{180}-	Kitayner M. *et al.* Mol. Cell 2006, 22, 741
2ATA_D	Cellular tumor antigen p53	Structural Basis of DNA Recognition by p53 Tetramers (complex II)	Apoptosis/DNA	2.20	-^{176}C-P-H-H-E^{180}-	Kitayner M. *et al.* Mol. Cell 2006, 22, 741
2AU3_A	DNA primase	Crosstalk between primase subunits can act to regulate primer synthesis in trans	Transferase	2.00	-^{35}C-P-F-H-P^{39}-	Corn J.E. *et al.* Mol. Cell 2005, 20, 391
2B8T_A	Thymidine kinase	Crystal structure of Thymidine Kinase from U.urealyticum in complex with thymidine	Transferase	2.00	-^{191}C-R-H-H-H^{195}-	Kosinska U. *et al.* FEBS Lett. 2005, 272, 6365
2B8T_B	Thymidine kinase	Crystal structure of Thymidine Kinase from U.urealyticum in complex with thymidine	Transferase	2.00	-^{191}C-R-H-H-H^{195}-	Kosinska U. *et al.* FEBS Lett. 2005, 272, 6365

2B8T_C	Thymidine kinase	Crystal structure of Thymidine Kinase from U.urealyticum in complex with thymidine	Transferase	2.00	-^{191}C-R-H-H-H^{195}-	Kosinska U. *et al.* FEBS Lett. 2005, 272, 6365
2B8T_D	Thymidine kinase	Crystal structure of Thymidine Kinase from U.urealyticum in complex with thymidine	Transferase	2.00	-^{191}C-R-H-H-H^{195}-	Kosinska U. *et al.* FEBS Lett. 2005, 272, 6365
2D5B_A	Methionyl-tRNA Synthetase	Crystal Structure of *Thermus Thermophilus* Methionyl tRNA synthetase Y225F Mutant obtained in the presence of PEG6000	Isomerase	1.80	-^{144}C-P-I-H-G^{148}-	Konno M. *et al.* To be published
2GEQ_A	Cellular tumor antigen p53	Crystal Structure of a p53 Core Dimer Bound to DNA	Transcription	2.30	-^{173}C-P-H-H-E^{177}-	Ho W.C. *et al.* J. Biol. Chem. 2006, 281, 20494
2GEQ_B	Cellular tumor antigen p53	Crystal Structure of a p53 Core Dimer Bound to DNA	Transcription	2.30	-^{173}C-P-H-H-E^{177}-	Ho W.C. *et al.* J. Biol. Chem. 2006, 281, 20494
2V0C_A	Aminoacyl-tRNA Synthetase	Leucyl L--tRNA Synthetase from *Thermus Thermophilus* complexed with a sulphamoyl amalogue Cof Leucyl-adenylate in the synthetic site and N adduct of AMP with 5-Fluoro-1,3-dihydro-1-hydroxy-2,1-Benzoxaborole (AN2690) Iin the editing state	Ligase	1.85	-^{176}C-W-R-H-E^{180}-	Rock F. *et al.* Science 2007, 316,1759
2V89_A	VDJ Recombination-Activating Protein 2	Crystal structure of RAG2-PHD finger in complex with H3K4me3 peptide at 1.1A resolution	Protein Binding	1.10	-^{1478}C-W-R-H-V^{1482}-	Matthews A.G.W *et al.* Nature 2007, 450,1106
2V89_B	VDJ Recombination-Activating Protein 2	Crystal structure of RAG2-PHD finger in complex with H3K4me3 peptide at 1.1A resolution	Protein Binding	1.10	-^{1478}C-W-R-H-V^{1482}-	Matthews A.G.W *et al.* Nature 2007, 450,1106
2YVR_A	Transcription intermediary factor 1-beta	Crystal structure of MS1043	Metal Binding Protein	1.80	-^{9}C-W-R-H-K^{13}-	Wang H. *et al.* To be published
2YVR_B	Transcription intermediary factor 1-beta	Crystal structure of MS1043	Metal Binding Protein	1.80	-^{9}C-W-R-H-K^{13}-	Wang H. *et al.* To be published
3D06_A	Cellular tumor antigen p53	Human p53 core domain with hot spot mutation R249S (I)	Transcription	1.20	-^{176}C-P-H-H-E^{180}-	Suad O. *et al.* J. Mol. Biol. 2009, 385, 249
3D0A_A	Cellular tumor antigen p53	Human p53 core domain with hot spot mutation R249S and second site suppressor mutation H168R in sequence-specific complex with DNA	Transcription	1.20	-^{176}C-P-H-H-E^{180}-	Suad O. *et al.* J. Mol. Biol. 2009, 385, 249

3D0A_B	Cellular tumor antigen p53	Human p53 core domain with hot spot mutation R249S and second site suppressor mutation H168R in sequence-specific complex with DNA	Transcription	1.20	$-^{176}$C-P-H-H-E^{180}-	Suad O. *et al.* J. Mol. Biol. 2009, 385, 249
3D0A_C	Cellular tumor antigen p53	Human p53 core domain with hot spot mutation R249S and second site suppressor mutation H168R in sequence-specific complex with DNA	Transcription	1.20	$-^{176}$C-P-H-H-E^{180}-	Suad O. *et al.* J. Mol. Biol. 2009, 385, 249
3D0A_D	Cellular tumor antigen p53	Human p53 core domain with hot spot mutation R249S and second site suppressor mutation H168R in sequence-specific complex with DNA	Transcription	1.20	$-^{176}$C-P-H-H-E^{180}-	Suad O. *et al.* J. Mol. Biol. 2009, 385, 24
3DDT_A	E3 ubiquitin-protein ligase TRIM63	Crystal structure of the B2 box from MuRF1 in dimeric state	Ligase	1.90	$-^{6}$C-K-E-H-E^{10}-	Mrosek M. *et al.* Biochemistry 2008, 47, 10730
3DDT_B	E3 ubiquitin-protein ligase TRIM63	Crystal structure of the B2 box from MuRF1 in dimeric state	Ligase	1.90	$-^{6}$C-K-E-H-E^{10}-	Mrosek M. *et al.* Biochemistry 2008, 47, 10730
3DDT_C	E3 ubiquitin-protein ligase TRIM63	Crystal structure of the B2 box from MuRF1 in dimeric state	Ligase	1.90	$-^{6}$C-K-E-H-E^{10}-	Mrosek M. *et al.* Biochemistry 2008, 47, 10730
3E2I_A	Thymidine kinase	Crystal structure of Thymidine Kinase from *S. aureus*	Transferase	2.01	$-^{183}$C-R-A-H-H^{187}-	Lam R. *et al.* To be Published
3EBE_A	Protein MCM10 homolog	Crystal structure of *xenopus laevis* replication initiation factor MCM10 internal domain	Replication	2.30	$-^{403}$C-Q-Y-H-V^{407}-	Warren E.M. *et al.* Structure 2008, 16, 1892
3EBE_B	Protein MCM10 homolog	Crystal structure of *xenopus laevis* replication initiation factor MCM10 internal domain	Replication	2.30	$-^{403}$C-Q-Y-H-V^{407}-	Warren E.M. *et al.* Structure 2008, 16, 1892
3EBE_C	Protein MCM10 homolog	Crystal structure of *xenopus laevis* replication initiation factor MCM10 internal domain	Replication	2.30	$-^{403}$C-Q-Y-H-V^{407}-	Warren E.M. *et al.* Structure 2008, 16, 1892
3EXJ_A	mouse p53 core domain	Crystal Structure of a p53 Core Tetramer Bound to DNA	Transcription	2.00	$-^{173}$C-P-H-H-E^{177}-	Malecka K.A. *et al.* Oncogene 2009, 28, 325
3EXJ_B	mouse p53 core domain	Crystal Structure of a p53 Core Tetramer Bound to DNA	Transcription	2.00	$-^{173}$C-P-H-H-E^{177}-	Malecka K.A. *et al.* Oncogene 2009, 28, 325
3EXL_A	mouse p53 core domain	Crystal Structure of a p53 Core Tetramer Bound to DNA	Transcription	2.20	$-^{173}$C-P-H-H-E^{180}-	Malecka K.A. *et al.* Oncogene 2009, 28, 325
3IGK_A	Cellular tumor antigen p53	Diversity in DNA recognition by p53 revealed by crystal structures with Hoogsteen base pairs (p53-DNA complex 2)	Transcription	1.70	$-^{176}$C-P-H-H-E^{180}-	Kitayner M. *et al.* Nat.Struct.Mol.Biol. 2010, 17, 423

3IGL_A	Cellular tumor antigen p53	Diversity in DNA recognition by p53 revealed by crystal structures with Hoogsteen base pairs (p53-DNA complex 1)	Transcription	1.80	-^{176}C-P-H-H-E^{180}-	Kitayner M. *et al.* Nat.Struct.Mol.Biol. 2010, 17, 423
3KMD_A	Cellular tumor antigen p53	Crystal structure of the p53 core domain bound to a full consensus site as a self-assembled tetramer	Transcription	2.15	-^{176}C-P-H-H-E^{180}-	Chen Y. *et al.* Structure 2010, 18, 246
3KMD_B	Cellular tumor antigen p53	Crystal structure of the p53 core domain bound to a full consensus site as a self-assembled tetramer	Transcription	2.15	-^{176}C-P-H-H-E^{180}-	Chen Y. *et al.* Structure 2010, 18, 246
3KMD_C	Cellular tumor antigen p53	Crystal structure of the p53 core domain bound to a full consensus site as a self-assembled tetramer	Transcription	2.15	-^{176}C-P-H-H-E^{180}-	Chen Y. . et al. Structure 2010, 18, 246
3KMD_D	Cellular tumor antigen p53	Crystal structure of the p53 core domain bound to a full consensus site as a self-assembled tetramer	Transcription	2.15	-^{176}C-P-H-H-E^{180}-	Chen Y. *et al.* Structure 2010, 18, 246
3KZ8_A	Cellular tumor antigen p53	Diversity in DNA recognition by p53 revealed by crystal structures with Hoogsteen base pairs (p53-DNA complex 3)	Transcription	1.91	-^{176}C-P-H-H-E^{180}-	Kitayner M. *et al.* Nat. Struct. Mol. Biol. 2010, 17, 423
3KZ8_B	Cellular tumor antigen p53	Diversity in DNA recognition by p53 revealed by crystal structures with Hoogsteen base pairs (p53-DNA complex 3)	Transcription	1.91	-^{176}C-P-H-H-E^{180}-	Kitayner M. *et al.* Nat.Struct.Mol.Biol. 2010, 17, 423
3MHJ_A	Tankyrase-2	Human tankyrase 2 - catalytic PARP domain in complex with 1-methyl-3-(trifluoromethyl)-5h-benzo[c][1,8]naphtyridine-6-one	Transferase	1.80	-^{1081}C-P-V-H-K^{1085}-	Wahlberg E. *et al.* Nat. Biotechnol. 2012, 30, 283
3MHJ_B	Tankyrase-2	Human tankyrase 2 - catalytic PARP domain in complex with 1-methyl-3-(trifluoromethyl)-5h-benzo[c][1,8]naphtyridine-6-one	Transferase	1.80	-^{1081}C-P-V-H-K^{1085}-	Wahlberg E. *et al.* Nat. Biotechnol. 2012, 30, 283
3MHS_A	Ubiquitin carboxyl-terminal hydrolase 8	Structure of the SAGA Ubp8/Sgf11/Sus1/Sgf73 DUB module bound to ubiquitin aldehyde	Hydrolase/ transcription regulator/protein binding	1.89	-^{273}C-I-V-H-T^{277}-	Samara N.L. *et al.* Science 2010, 328, 1025
3NY1_A	E3 ubiquitin-protein ligase UBR1	Structure of the ubr-box of the UBR1 ubiquitin ligase	Ligase	2.09	-^{163}C-V-N-H-E^{167}-	Matta-Camacho E. *et al.* Nat. Struct. Mol. Biol. 2010, 17, 1182
3NY1_B	E3 ubiquitin-protein ligase UBR1	Structure of the ubr-box of the UBR1 ubiquitin ligase	Ligase	2.09	-^{163}C-V-N-H-E^{167}-	Matta-Camacho E. *et al.* Nat. Struct. Mol. Biol. 2010, 17, 1182
3OHR_A	Putative fructokinase	Crystal structure of fructokinase from *bacillus subtilis* complexed with ADP	Transferase	1.66	-^{168}C-P-Y-H-G^{172}-	Nocek B. *et al.* J. Mol. Biol. 2011, 406,

3PLW_A	Recombination enhancement function protein	Ref protein from P1 bacteriophage	Hydrolase	1.40	-^{99}C-P-Y-H-H^{103}-	Gruenig M.C. *et al.* J. Biol. Chem. 2011, 286, 8240
3Q01_A	Cellular tumor antigen p53	An induced fit mechanism regulates p53 DNA binding kinetics to confer sequence specificity	Antitumor protein	2.10	-^{176}C-P-H-H-E^{180}-	Petty T.J. *et al.* Embo J. 2011, 30, 2167
3Q01_B	Cellular tumor antigen p53	An induced fit mechanism regulates p53 DNA binding kinetics to confer sequence specificity	Antitumor protein	2.10	-^{176}C-P-H-H-E^{180}-	Petty T.J. *et al.* Embo J. 2011, 30, 2167
3Q05_A	Cellular tumor antigen p53	An induced fit mechanism regulates p53 DNA binding kinetics to confer sequence specificity	Antitumor protein	2.40	-^{176}C-P-H-H-E^{180}-	Petty T.J. *et al.* Embo J. 2011, 30, 2167
3Q05_B	Cellular tumor antigen p53	An induced fit mechanism regulates p53 DNA binding kinetics to confer sequence specificity	Antitumor protein	2.40	-^{176}C-P-H-H-E^{180}-	Petty T.J. *et al.* Embo J. 2011, 30, 2167
3Q05_C	Cellular tumor antigen p53	An induced fit mechanism regulates p53 DNA binding kinetics to confer sequence specificity	Antitumor protein	2.40	-^{176}C-P-H-H-E^{180}-	Petty T.J. *et al.* Embo J. 2011, 30, 2167
3Q05_D	Cellular tumor antigen p53	An induced fit mechanism regulates p53 DNA binding kinetics to confer sequence specificity	Antitumor protein	2.40	-^{176}C-P-H-H-E^{180}-	Petty T.J. *et al.* Embo J. 2011, 30, 2167
3Q1D_A	Tripartite motif-containing protein 54	The B-box domain of Trim54	Ligase	2.15	-^{126}C-E-E-H-E^{130}-	Walker J.R. *et al.* To be published
3RAY_A	PR domain-containing protein 11	Crystal structure of Methyltransferase domain of human PR domain-containing protein 11	Transcription	1.73	-^{43}C-P-N-H-G^{47}-	Dong A. *et al.* To be published
3RYM_A	Amicyanin	Structure of Oxidized M98K mutant of Amicyanin	Electron Transport	1.70	-^{92}C-P-N-H-P^{96}-	Sukumar N. *et al.* J. Inorg. Chem. 2011, 105, 1638
3RYM_B	Amicyanin	Structure of Oxidized M98K mutant of Amicyanin	Electron Transport	1.70	-^{92}C-P-N-H-P^{96}-	Sukumar N. *et al.* J. Inorg. Chem. 2011, 105, 1638
3RYM_C	Amicyanin	Structure of Oxidized M98K mutant of Amicyanin	Electron Transport	1.70	-^{92}C-P-N-H-P^{96}-	Sukumar N. *et al.* J. Inorg. Chem. 2011, 105, 1638
3RYM_D	Amicyanin	Structure of Oxidized M98K mutant of Amicyanin	Electron Transport	1.70	-^{92}C-P-N-H-P^{96}-	Sukumar N. *et al.* J. Inorg. Chem. 2011, 105, 1638
3VRH_A	Putative uncharacterized protein PH0300	Crystal structure of ph0300	RNA Binding Protein	2.10	-^{22}C-E-E-H-F^{26}-	Nakagawa H. *et al.* Proteins 2013, 81,

4C1Q_A	Histone-Lysine N-methyltransferase PRDM9	Crystal structure of the PRDM9 SET domain in complex with H3K4me2 and AdoHcy.	Transferase	2.30	-^{216}C-P-N-H-G^{220}-	Wu H. *et al.* Cell Rep. 2013, 5, 13
4C1Q_B	Histone-Lysine N-methyltransferase PRDM9	Crystal structure of the PRDM9 SET domain in complex with H3K4me2 and AdoHcy.	Transferase	2.30	-^{216}C-P-N-H-G^{220}-	Wu H. *et al.* Cell Rep. 2013, 5, 13
4GNE_A 2	Histone-lysine N-methyltransferase NSD3	Crystal Structure of NSD3 tandem PHD5-C5HCH domains complexed with H3 peptide 1-7	Transferase	1.47	-^{1405}C-S-E-H-E^{1409}-	He C. *et al.* J. Biol. Chem. 2013, 288, 4692
4HCG_A	Cupredoxin 1	Uncharacterized Cupredoxin-like Domain Protein Cupredoxin_1 with Zinc bound from *Bacillus anthracis*	Oxidoreductase	1.85	-^{113}C-R-Y-H-L^{117}-	Kim Y. *et al.* To be published
4HCG_B	Cupredoxin 1	Uncharacterized Cupredoxin-like Domain Protein Cupredoxin_1 with Zinc bound from *Bacillus anthracis*	Oxidoreductase	1.85	-^{113}C-R-Y-H-L^{117}-	Kim Y. *et al.* To be published
4HJE_A	Cellular tumor antigen p53	Crystal structure of p53 core domain in complex with DNA	Transcription	1.91	-^{176}C-P-H-H-E^{180}-	Chen Y. *et al.* Nucleic Acids Res. 2013, 41, 8368
4HJE_B	Cellular tumor antigen p53	Crystal structure of p53 core domain in complex with DNA	Transcription	1.91	-^{176}C-P-H-H-E^{180}-	Chen Y. *et al.* Nucleic Acids Res. 2013, 41, 8368
4HJE_C	Cellular tumor antigen p53	Crystal structure of p53 core domain in complex with DNA	Transcription	1.91	-^{176}C-P-H-H- E^{180}-	Chen Y. *et al.* Nucleic Acids Res. 2013, 41, 8368
4HJE_D	Cellular tumor antigen p53	Crystal structure of p53 core domain in complex with DNA	Transcription	1.91	-^{176}C-P-H-H^{-}E^{180}-	Chen Y. *et al.* Nucleic Acids Res. 2013, 41, 8368
4IBU_A	Cellular tumor antigen p53	Human p53 core domain with hot spot mutation R273C and second-site suppressor mutation T284R in sequence-specific complex with DNA	DNA Binding Protein	1.70	-^{176}C-P-H-H^{-}E^{180}-	Eldar A. *et al.* Nucleic Acids Res. 2013, 41, 8748
4IBU_B	Cellular tumor antigen p53	Human p53 core domain with hot spot mutation R273C and second-site suppressor mutation T284R in sequence-specific complex with DNA	DNA Binding Protein	1.70	-^{176}C-P-H-H-E^{180}-	Eldar A. *et al.* Nucleic Acids Res. 2013, 41, 8748
4IBU_C	Cellular tumor antigen p53	Human p53 core domain with hot spot mutation R273C and second-site suppressor mutation T284R in sequence-specific complex with DNA	DNA Binding Protein	1.70	-^{176}C-P-H-H-E^{180}-	Eldar A. *et al.* Nucleic Acids Res. 2013, 41, 8748
4IBU_D	Cellular tumor antigen p53	Human p53 core domain with hot spot mutation R273C and second-site suppressor mutation	DNA Binding Protein	1.70	-^{176}C-P-H-H-E^{180}-	Eldar A. *et al.* Nucleic Acids Res.

		T284R in sequence-specific complex with DNA				2013, 41, 8748
4IBV_A	Cellular tumor antigen p53	Human p53 core domain with hot spot mutation R273C and second-site suppressor mutation S240R in sequence-specific complex with DNA	DNA Binding Protein	2.10	-^{176}C-P-H-H-E^{180}-	Eldar A. *et al.* Nucleic Acids Res. 2013, 41, 8748
4IBW_A	Cellular tumor antigen p53	Human p53 core domain with hot spot mutation R273H and second-site suppressor mutation T284R in sequence-specific complex with DNA	DNA Binding Protein	1.79	-^{176}C-P-H-H-E^{180}-	Eldar A. *et al.* Nucleic Acids Res. 2013, 41, 8748
4IJD_A	Histone-lysine N-methyltransferase PRDM9	Crystal structure of methyltransferase domain of human PR domain-containing protein 9	Transferase	2.15	-^{216}C-A-A-H-G^{220}-	Dombrovski L. *et al.* To be published
4IJD_B	Histone-lysine N-methyltransferase PRDM9	Crystal structure of methyltransferase domain of human PR domain-containing protein 9	Transferase	2.15	-^{216}C-A-A-H-G^{220}-	Dombrovski L. *et al.* To be published
4IXJ_A	Fimbrial protein (Pilin)	The structure of PilJ, a Type IV pilin from *Clostridium difficile*	Cell Adhesion	1.98	-^{111}C-T-K-H-P^{115}-	Piepenbrink K.H. *et al.* J. Biol. Chem. 2014, 289, 4334
4IXJ_B	Fimbrial protein (Pilin)	The structure of PilJ, a Type IV pilin from *Clostridium difficile*	Cell Adhesion	1.98	-^{111}C-T-K-H-P^{115}-	Piepenbrink K.H. *et al.* J. Biol. Chem. 2014, 289, 4334
4LJO_A	E3 ubiquitin-protein ligase RNF31	Structure of an active ligase (HOIP)/ubiquitin transfer complex	Ligase	1.56	-^{998}C-Q-A-H-Y^{1002}-	Stieglitz B. *et al.* Nature 2013, 503, 422
4NN2_A	PHD finger protein 6	Protein Crystal Structure of Human Borjeson-Forssman-Lehmann Syndrome Associated Protein PHF6	Transcription	1.47	-^{326}C-K-N-H-S^{330}-	Liu Z. *et al.* J. Biol. Chem. 2014
4NN2_B	PHD finger protein 6	Protein Crystal Structure of Human Borjeson-Forssman-Lehmann Syndrome Associated Protein PHF6	Transcription	1.47	-^{326}C-K-N-H-S^{330}-	Liu Z. *et al.* J. Biol. Chem. 2014
4QO1_B	Cellular tumor antigen p53	p53 DNA binding domain in complex with Nb139	Apoptosis transcription	1.92	-^{176}C-P-H-H-E^{180}-	Bethuyne J. *et al.* Nucleic Acids Res. 2014, 42, 12928
4RH4_A	Pseudoazurin	Zinc-substituted pseudoazurin solved by S/Zn-SAD phasing	Metal Binding Protein	1.60	-^{78}C-T-P-H-Y^{82}-	Gessmann R. *et al.* Acta Crystallogr. F Struct. Biol. Commun. 2015, 71, 19
4TOR_A	Tankyrase-1	Crystal structure of Tankyrase 1 with IWR-8	Transferase	1.50	-^{1234}C-P-T-H-K^{1238}-	Kulak O. *et al.* Mol. Cell. Biol. 2015, 35, 2425

4TOR_B	Tankyrase-1	Crystal structure of Tankyrase 1 with IWR-8	Transferase	1.50	-^{1234}C-P-T-H-K^{1238}-	Kulak O. *et al.* Mol. Cell. Biol. 2015, 35, 2425
4TOR_C	Tankyrase-1	Crystal structure of Tankyrase 1 with IWR-8	Transferase	1.50	-^{1234}C-P-T-H-K^{1238}-	Kulak O. *et al.* Mol. Cell. Biol. 2015, 35, 2425
4TOR_D	Tankyrase-1	Crystal structure of Tankyrase 1 with IWR-8	Transferase	1.50	-^{1234}C-P-T-H-K^{1238}-	Kulak O. *et al.* Mol. Cell. Biol. 2015, 35, 2425
4YBG_A	Protein maelstrom	Crystal structure of the MAEL domain of *Drosophila melanogaster* Maelstrom	Hydrolase	1.60	-^{288}C-Q-Y-H-E^{292}-	Matsumoto N. *et al.* Cell. Rep. 2015, 11, 366
5AF0_A	Maelstrom	MAEL domain from Bombyx mori Maelstrom	Unknown Protein	2.40	-^{290}C-E-R-H-E^{294}-	Chen K. *et al.* RNA 2015, 21, 833
5AF0_B	Maelstrom	MAEL domain from Bombyx mori Maelstrom	Unknown Protein	2.40	-^{290}C-E-R-H-E^{294}-	Chen K. *et al.* RNA 2015, 21, 833
5AF0_C	Maelstrom	MAEL domain from Bombyx mori Maelstrom	Unknown Protein	2.40	-^{290}C-E-R-H-E^{294}-	Chen K. *et al.* RNA 2015, 21, 833
5AF0_D	Maelstrom	MAEL domain from Bombyx mori Maelstrom	Unknown Protein	2.40	-^{290}C-E-R-H-E^{294}-	Chen K. *et al.* RNA 2015, 21, 833
5C5R_A	Tankyrase-2	Crystal structure of Human Tankyrase-2 in complex with a pyranopyridone inhibitor	Transferase/ Transferase inhibitor	1.55	-^{1081}C-P-V-H-K^{1085}-	De Vincente J. *et al.* Acs Med.Chem.Lett. 2015, 6, 1019
5C5R_B	Tankyrase-2	Crystal structure of Human Tankyrase-2 in complex with a pyranopyridone inhibitor	Transferase/ Transferase inhibitor	1.55	-^{1081}C-P-V-H-K^{1085}-	De Vincente J. *et al.* Acs Med.Chem.Lett. 2015, 6, 1019
5EIU_A	TRIM protein-E3 ligase Chimera	Mini TRIM5 B-box 2 dimer C2 crystal form	Ligase	1.91	-^{97}C-A-R-H-G^{101}-	Wagner J.M. *et al.* Elife, 2016, 5
5FV3_A	Lysine-specific demethylase 5B	Crystal structure of human JARID1B construct c2 in complex with N- Oxalylglycine	Oxidoreductase	2.37	-^{715}C-L-H-H-V^{719}-	Johansson C. *et al.*, Nat. Chem. Biol. 2016, 12, 539
5O6C_A	E3 ubiquitin-protein ligase MYCBP2	Crystal structure of a threonine-selective RCR E3 ligase	Ligase	1.75	-^{4549}C-P-K-H-G^{4553}-	Pao K.C. *et al.* Nature 2018, 556, 381
5OLM_A	E3 ubiquitin-protein ligase TRIM21	Intracellular antibody signalling is regulated by phosphorylation of the Fc receptor TRIM21	Antivirial protein	1.95	-^{92}C-A-V-H-G^{96}-	Dickson C. *et al.* Elife 2018, 7
5YKN_A	Probable lysine-specific demethylase JMJ14	Crystal structure of Arabidopsis thaliana JMJ14 catalytic domain	Gene regulation	2.30	-^{542}C-L-I-H-A^{546}-	Yang Z. *et al.* Plant Cell 2017, 30,167
5ZTB_A	Sulfur carrier protein TtuB	Structure of Sulfurtransferase	RNA binding protein/	2.20	-^{22}C-R-E-H-Y^{26}-	Chen M. *et al.* Commun Biol 2020, 3,

			Transferase			168
6LND_K	Transposition protein TniQ	Crystal structure of transposition protein TniQ	DNA binding protein	2.00	-^{150}C-H-S-H-N^{154}-	Wang B. *et al.* Cell Res 2020, 30, 185
6MM1_A	Histone-lysine N-methyltransferase EHMT2	Structure of the cysteine-rich region from human EHMT2	Transferase	1.90	-^{65}C-E-T-H-R^{69}-	Kerchner K.M. *et al.* To be published
6U04_A	Histone H3.3, BRPF1, Peregrin	Crystal structure of human BRPF1 PZP bound to histone H3 tail	Transcription	2.20	-^{444}C-D-I-H-T^{448}-	Klein B.J. *et al.* Structure 2020, 28, 105
6VHF_A	PHD-type domain-containing protein	Crystal structure of RbBP5 interacting domain of Cfp1	Peptide binding protein	2.31	-^{456}C-K-P-H-N^{460}-	Yang Y. *et al.* Nucleic Acids Res 2020, 48, 421
6ZSL_B	SARS-CoV-2 helicase NSP13	Crystal structure of the SARS-CoV-2 helicase at 1.94 Angstrom resolution	Hydrolase	1.94	-^{72}C-K-S-H-K^{76}-	Newman J.A. *et al.* To be published
7D2T_B	Ras suppressor protein 1	Crystal structure of Rsu1/PINCH1_LIM45C complex	Protein binding	2.20	-^{240}C-E-T-H-Y^{244}-	Yang H. *et al.* Elife 2020, 10

[a] When the pdb contains more than one chain, the identifier of the chain containing the C-X_1-X_2-H-X_3 motif is specified.

Table S3. Non-redundant C-X_1-X_2-H-X_3 motif and their classification (X_1, X_2, X_3 = any amino acid). The amino acids are indicated as single letter codes.

Cluster 1	Cluster 2	Cluster 3	Cluster 4	Outlier 5	Outlier 7
CRSHT	CPIHG	CEHHL	CWRHE	CPFHP	CKPHN
CPHHE	CPYHG	CPVHK	CNVHK		
CQEHS	CPNHG	CPTHK	CKEHE		
CRHHH	CRYHL		CEEHE		
CNEHV	CAAHG		CTPHP		
CRAHH	CTKHP				
CQYHV	CTPHY				
CIVHT	CARHG				
CVNHE	CPKHG				
CRWHH	CAVHG				
CEEHF	CHSHN				
CKDHE					
CQAHY					
CKNHS					
CQYHE					
CERHE					
CLHHV					
CLIHA					
CREHY					
CETHR					
CDIHT					
CKSHK					
CETHY					

Table S4. Database entries: conformational and metal coordination parameters for each C-X_1-X_2-H-X_3 motif (X_1, X_2, X_3 = any amino acid). Angles are expressed in degrees and distances are expressed in angstroms. The centroid of each cluster is highlighted in bold.

PDB code[a]	C-X_1-X_2-H-X_3 motif	ϕ_{i+1}	ψ_{i+1}	ϕ_{i+2}	ψ_{i+2}	ϕ_{i+3}	ψ_{i+3}	χ_1 Cys	χ_1 His	d(Sγ-Zn)	d (Nδ-Zn)	Sγ-Zn-Nδ Angle	Cβ-Sγ-Zn Angle	QR-Nδ-Zn Angle
								**Cluster 1**						
1T4W_A	CRSHT	-58.4	-48.6	-67.5	-33.7	-77.9	-28.5	74.4	-82.0	2.4	2.0	103.3	108.0	169.0
1TSR _A	CPHHE	-66.4	-44.7	-46.6	-53.8	-62.6	-53.2	73.8	-67.0	2.3	2.0	107.2	109.7	172.3
1TSR_B	CPHHE	-54.8	-47.1	-56.8	-61.8	-57.7	-32.0	74.6	-71.5	2.3	2.1	103.0	111.0	165.7
1TSR_C	CPHHE	-64.2	-34.7	-53.7	-70.4	-57.0	-18.5	62.4	-89.1	2.4	2.1	101.9	110.2	169.0
1TUP_A	CPHHE	-66.8	-43.6	-47.5	-53.4	-62.4	-53.4	74.1	-66.2	2.3	2.0	107.6	110.0	172.5
1TUP_B	CPHHE	-55.8	-47.3	-56.6	-61.7	-57.9	-31.7	74.3	-71.6	2.3	2.1	102.5	110.7	165.7
1TUP_C	CPHHE	-64.3	-34.8	-53.4	-71.5	-55.6	-17.5	63.0	-88.6	2.4	2.1	101.6	109.1	169.0
1VFY_A	CQEHS	-57.9	-42.1	-59.8	-29.7	-105.0	15.9	68.1	-67.1	2.3	2.1	96.1	114.0	174.2
1YCS_A	CPHHE	-42.9	-49.8	-65.5	-49.6	-62.0	-40.6	70.4	-87.9	2.4	1.9	103.3	112.3	173.2
2AC0_A	CPHHE	-56.2	-41.0	-59.4	-52.7	-68.5	-35.6	68.7	-77.7	2.3	1.9	104.1	114.6	176.3
2AC0_B	CPHHE	-57.8	-37.6	-63.2	-55.5	-75.1	-27.1	72.5	-80.1	2.3	2.1	102.7	109.5	176.8
2AC0_C	CPHHE	-58.7	-32.3	-69.1	-53.2	-72.8	-31.0	72.3	-79.8	2.4	2.1	100.5	112.5	172.6
2AC0_D	CPHHE	-57.0	-35.3	-66.3	-53.0	-67.8	-33.0	73.4	-79.1	2.4	2.0	101.1	107.6	178.5
2AHI_A	CPHHE	-57.6	-37.3	-59.2	-54.9	-71.9	-29.4	69.6	-77.5	2.4	2.0	102.8	113.7	174.2
2AHI_B	CPHHE	-60.5	-38.6	-64.7	-54.9	-67.0	-30.9	74.6	-75.2	2.4	2.1	102.4	110.8	174.0
2AHI_C	CPHHE	-54.4	-36.0	-67.8	-53.6	-70.3	-29.5	71.5	-76.5	2.4	2.1	102.8	110.5	176.6
2AHI_D	CPHHE	-54.3	-36.8	-67.8	-50.1	-69.4	-32.8	71.4	-77.1	2.4	2.0	102.6	109.4	174.7
2ATA_A	CPHHE	-60.1	-32.9	-70.6	-49.9	-71.1	-30.8	70.5	-74.5	2.4	1.9	102.4	111.2	173.6

2ATA_B	CPHHE	-54.9	-40.5	-57.1	-54.6	-74.4	-32.4	67.7	-75.3	2.2	2.2	101.2	116.8	171.2
2ATA_C	CPHHE	-57.2	-31.0	-72.3	-55.2	-68.8	-28.2	75.3	-77.1	2.4	2.0	104.2	109.2	171.6
2ATA_D	CPHHE	-53.8	-38.2	-62.6	-54.6	-67.6	-30.9	71.8	-78.1	2.4	2.0	98.5	111.4	172.0
2B8T_A	CRHHH	-52.1	-48.0	-57.3	-35.2	-101.0	15.3	63.7	-65.6	2.3	2.1	94.1	117.6	165.7
2B8T_B	CRHHH	-57.6	-41.1	-62.8	-34.4	-95.5	8.4	64.8	-69.9	2.4	2.1	100.5	116.6	170.2
2B8T_C	CRHHH	-54.8	-48.4	-61.1	-29.1	-101.4	9.2	66.5	-67.1	2.4	2.1	88.4	111.4	163.4
2B8T_D	CRHHH	-53.9	-45.0	-61.9	-36.0	-94.4	11.3	65.8	-70.6	2.3	2.1	93.7	115.0	170.7
2GEQ_A	CPHHE	-56.6	-30.2	-68.9	-53.6	-69.2	-27.1	72.9	-75.1	2.3	2.1	109.1	111.4	172.2
2GEQ_B	CPHHE	-54.5	-40.5	-58.9	-53.1	-71.9	-27.7	68.5	-78.6	2.3	2.1	105.8	113.2	170.4
2V89_A	CNEHV	-61.4	-28.5	-75.6	-29.1	-115.9	-16.9	78.8	-64.6	2.3	2.0	101.7	108.9	167.0
2V89_B	CNEHV	-62.8	-28.9	-71.7	-27.2	-114.0	-5.6	75.4	-64.2	2.3	2.1	101.2	108.8	166.5
3D06_A	CPHHE	-54.6	-41.8	-60.8	-49.7	-72.7	-31.6	71.6	-79.5	2.3	2.0	102.2	110.4	173.9
3D0A_A	CPHHE	-54.9	-37.9	-60.6	-56.8	-69.2	-33.1	70.0	-80.9	2.3	2.0	103.8	114.5	170.8
3D0A_B	CPHHE	-57.1	-34.6	-64.5	-55.5	-69.5	-30.0	69.2	-76.0	2.4	2.0	102.8	112.9	173.4
3D0A_C	CPHHE	-54.1	-39.1	-62.9	-52.8	-71.6	-29.7	73.4	-76.8	2.3	1.8	103.4	108.9	167.9
3D0A_D	CPHHE	-57.1	-31.1	-69.5	-53.9	-66.7	-33.2	76.3	-74.3	2.3	2.1	103.6	107.7	177.3
3E2I_A	CRAHH	-50.8	-42.0	-65.5	-21.0	-117.0	4.9	64.4	-63.8	2.2	2.3	88.8	119.4	163.2
3EBE_A	CQYHV	-46.3	-54.3	-60.3	-40.7	-83.8	-21.2	71.6	-72.5	2.2	2.0	102.2	113.7	165.5
3EBE_B	CQYHV	-71.6	-22.9	-68.8	-33.0	-111.0	-5.1	72.4	-70.2	2.4	2.0	107.5	112.2	168.8
3EBE_C	CQYHV	-60.6	-27.2	-80.5	-18.0	-114.6	-10.9	69.9	-72.5	2.3	2.0	100.3	109.9	175.8
3EXJ_A	CPHHE	-53.7	-39.5	-67.6	-58.3	-63.7	-31.9	69.3	-72.6	2.4	2.2	102.1	111.6	175.4

3EXJ_B	CPHHE	-55.7	-38.6	-68.5	-53.5	-69.5	-26.0	70.0	-72.1	2.3	2.1	102.0	110.5	173.3
3EXL_A	CPHHE	-53.7	-32.6	-70.0	-53.8	-68.6	-23.6	69.2	-76.0	2.3	2.2	97.0	110.0	161.6
3IGK_A	CPHHE	-64.5	-33.9	-63.4	-52.8	-73.9	-19.2	79.5	-73.7	2.3	2.1	104.8	108.9	177.7
3IGL_A	CPHHE	-61.3	-36.4	-59.7	-59.2	-66.6	-25.9	75.4	-69.2	2.3	2.0	104.4	109.8	177.4
**3KMD_A**	**CPHHE**	**-60.8**	**-37.1**	**-66.0**	**-52.4**	**-70.2**	**-27.7**	**72.2**	**-77.3**	**2.4**	**2.0**	**103.1**	**110.6**	**174.6**
3KMD_B	CPHHE	-61.9	-38.0	-65.9	-56.0	-63.9	-34.5	70.8	-72.9	2.4	2.1	104.3	110.9	179.4
3KMD_C	CPHHE	-60.1	-34.3	-67.2	-53.8	-68.8	-30.7	75.2	-72.9	2.4	2.0	102.6	109.1	175.1
3KMD_D	CPHHE	-59.3	-34.6	-67.6	-51.2	-72.2	-30.8	76.1	-78.2	2.3	2.2	99.9	107.8	172.4
3KZ8_A	CPHHE	-65.9	-33.7	-62.5	-55.5	-71.5	-17.7	73.6	-67.1	2.3	2.0	106.5	112.8	172.7
3KZ8_B	CPHHE	-65.7	-33.9	-63.2	-55.3	-71.7	-17.9	74.6	-67.9	2.3	2.0	106.5	111.9	172.8
3MHS_A	CIVHT	-61.1	-37.4	-64.4	-46.7	-83.3	-10.5	79.5	-72.1	2.3	2.1	108.8	107.7	173.8
3NY1_A	CVNHE	-65.2	-38.5	-60.5	-42.5	-99.3	-3.0	69.8	-56.9	2.3	2.1	97.7	111.6	172.9
3NY1_B	CVNHE	-56.7	-31.3	-75.4	-28.1	-108.8	-0.2	72.3	-60.4	2.3	2.1	103.9	111.4	168.3
3PLW_A	CRWHH	-61.3	-32.7	-80.6	-20.9	-95.7	-10.4	70.0	-90.5	2.4	2.1	105.3	106.4	174.2
3Q01_A	CPHHE	-60.5	-44.5	-51.1	-50.1	-75.4	-30.6	69.4	-82.8	2.4	2.2	100.5	108.9	171.2
3Q01_B	CPHHE	-59.0	-44.8	-49.3	-53.7	-71.6	-34.4	70.1	-81.8	2.4	2.2	104.3	109.0	164.3
3Q05_A	CPHHE	-54.3	-39.8	-58.7	-58.0	-70.0	-20.6	71.2	-78.4	2.4	2.4	102.4	113.5	171.6
3Q05_B	CPHHE	-58.7	-39.5	-63.1	-57.2	-70.8	-14.9	83.4	-77.6	2.7	2.4	103.1	99.1	174.6
3Q05_C	CPHHE	-54.8	-37.4	-66.2	-52.9	-73.1	-26.9	65.3	-75.2	2.3	2.2	109.9	120.8	176.0
3Q05_D	CPHHE	-54.2	-40.0	-58.4	-57.6	-70.3	-22.1	70.9	-78.4	2.4	2.3	104.4	116.0	171.8
3VRH_A	CEEHF	-52.0	-50.7	-71.6	-38.3	-74.3	-25.4	74.4	-81.5	2.5	2.2	95.6	102.4	162.2

4GNE_A2	CKDHE	-59.4	-28.8	-77.9	-26.9	-118.4	5.1	72.1	-60.8	2.3	2.1	98.7	111.9	168.8
4HJE_A	CPHHE	-58.5	-36.8	-68.0	-52.6	-68.2	-31.1	74.0	-77.6	2.4	2.1	103.1	109.7	178.9
4HJE_B	CPHHE	-57.9	-36.9	-68.2	-51.3	-70.0	-30.7	74.1	-77.0	2.3	2.1	102.9	107.1	179.4
4HJE_C	CPHHE	-57.7	-36.6	-67.2	-53.3	-68.8	-28.9	74.5	-77.8	2.3	2.1	103.9	109.0	178.8
4HJE_D	CPHHE	-57.8	-38.9	-65.9	-53.1	-68.6	-29.0	73.1	-77.4	2.3	2.1	103.3	109.6	179.6
4IBU_A	CPHHE	-57.5	-35.8	-64.8	-53.0	-67.9	-31.7	73.1	-79.4	2.3	2.0	106.5	110.8	173.6
4IBU_B	CPHHE	-58.3	-33.5	-68.4	-54.1	-67.9	-30.8	74.8	-77.9	2.4	2.1	105.3	107.4	175.7
4IBU_C	CPHHE	-58.4	-35.1	-64.8	-55.3	-67.7	-29.8	75.6	-79.0	2.3	2.1	106.6	108.1	178.1
4IBU_D	CPHHE	-54.0	-37.5	-66.3	-52.1	-67.8	-33.8	73.0	-81.2	2.3	2.0	106.4	111.3	177.2
4IBV_A	CPHHE	-65.9	-29.5	-62.8	-55.1	-71.9	-23.0	80.6	-69.9	2.3	2.1	105.6	108.1	176.8
4IBW_A	CPHHE	-61.0	-35.3	-61.8	-52.8	-73.0	-29.3	76.4	-73.6	2.3	2.1	103.4	109.8	177.0
4LJO_A	CQAHY	-57.5	-48.1	-67.6	-43.0	-73.3	-31.9	65.2	-77.4	2.2	2.1	99.7	110.6	175.0
4NN2_A	CKNHS	-59.5	-26.7	-79.5	-18.0	-121.0	23.9	80.0	-68.3	2.3	2.1	103.8	107.6	175.7
4NN2_B	CKNHS	-59.4	-24.2	-83.8	-14.6	-125.6	13.7	79.8	-66.2	2.3	2.1	101.5	106.2	170.1
4QO1_B	CPHHE	-53.8	-44.7	-55.0	-51.9	-75.5	-34.5	78.1	-82.7	2.4	2.0	104.3	107.4	174.6
4YBG_A	CQYHE	-64.8	-40.2	-60.2	-42.9	-77.5	-23.9	72.1	-83.3	2.3	2.0	103.6	109.2	175.0
5AFO_A	CERHE	-54.3	-45.5	-58.2	-53.7	-63.5	-40.8	80.1	-79.8	2.4	2.1	78.4	105.0	148.2
5AF0_C	CERHE	-50.6	-43.3	-59.5	-55.4	-63.6	-47.6	79.3	-82.5	2.4	2.2	87.7	93.6	161.6
5AF0_D	CERHE	-51.8	-42.8	-58.6	-58.0	-63.2	-44.1	78.9	-83.0	2.3	2.1	91.1	101.2	163.4
5FV3_A	CLHHV	-76.1	-3.3	-85.8	-16.3	-130.6	48.6	74.3	-62.1	2.3	2.3	99.7	110.6	170.4
5YKN_A	CLIHA	-65.3	-20.2	-72.0	-36.7	-108.4	28.9	73.4	-53.4	2.3	2.1	105.6	110.8	147.3

5ZTB_A†	CREHY	-59.9	-56.0	-67.3	-33.8	-80.4	-28.7	65.1	-71.9	2.2	2.2	120.1	112.2	114.2
6MM1_A	CETHR	-63.7	-34.6	-64.3	-41.8	-79.3	-27.6	73.7	-85.5	2.3	2.0	106.6	111.3	169.9
6U04_A	CDIHT	-72.2	-15.2	-71.0	-30.6	-116.1	13.4	77.9	-68.4	2.4	2.0	102.4	113.1	174.9
6ZSL_B	CKSHK	-66.3	-36.7	-71.8	-33.9	-109.2	24.1	71.7	-50.0	2.5	2.0	81.9	105.8	159.1
7D2T_B	CETHY	-56.9	-46.7	-63.2	-39.9	-82.4	-27.0	71.9	-74.8	2.3	2.1	97.5	106.6	159.9
Average		-59	-37	-65	-47	-79	- 21	72	-75	2.34	2.08	102	110	171
STD		(5)	(8)	(7)	(12)	(18)	(19)	(4)	(8)	(0.07)	(0.09)	(6)	(4)	(9)
							**Cluster 2**							
2D5B _A	CPIHG	-62.3	-9.6	-105.6	-52.6	-93.7	-8.4	173.7	-59.0	2.3	2.1	111.5	114.2	156.7
3OHR_A	CPYHG	-65.1	-26.2	-84.7	-44.0	-118.2	-12.7	178.5	-48.4	2.3	2.0	106.7	103.6	175.1
3RAY_A	CPNHG	-67.5	-6.0	-121.6	-21.3	-122.6	-5.7	175.6	-66.2	2.3	2.4	118.9	129.5	174.0
4C1Q_A	CPNHG	-64.7	-24.3	-89.5	-44.1	-108.3	-0.8	-179.0	-64.3	2.3	2.5	107.1	109.9	138.4
4C1Q_B	CPNHG	-62.1	-28.5	-87.7	-44.9	-108.2	-1.2	-179.4	-64.7	2.3	2.3	112.4	107.4	139.3
4HCG_A	CRYHL	-64.1	-23.8	-84.8	-25.2	-132.2	15.2	170.9	-60.0	2.3	2.0	110.1	99.4	175.1
4HCG_B	CRYHL	-63.6	-27.1	-81.6	-26.0	-129.1	15.8	169.0	-60.6	2.2	2.0	110.7	101.2	175.6
**4IJD_A**	**CAAHG**	**-56.0**	**-30.1**	**-91.5**	**-33.9**	**-119.1**	**-4.7**	**173.9**	**-61.9**	**2.3**	**2.2**	**111.6**	**115.0**	**168.3**
4IJD_B	CAAHG	-57.6	-38.5	-80.5	-45.9	-114.9	-0.6	-176.4	-56.1	2.4	2.2	120.9	106.1	161.4
4IXJ_A†	CTKHP	-61.0	-17.2	-106.8	-3.7	-138.1	89.4	171.7	-74.2	2.3	2.3	123.9	108.7	178.3
4IXJ_B†	CTKHP	-72.0	-18.1	-100.6	-20.1	-128.5	93.9	167.8	-67.6	2.4	2.2	123.4	106.8	170.4
4RH4_A	CTPHY	-50.8	-53.8	-63.5	-34.6	-105.1	15.0	167.8	-60.2	2.2	2.0	111.3	100.7	179.6
5EIU_A	CARHG	-60.4	-29.4	-96.6	-42.0	-107.3	-13.6	-177.1	-55.7	2.3	2.0	106.5	102.9	174.7
5O6C_A	CPKHG	-55.0	-35.3	-84.2	-43.4	-114.4	-2.3	-177.1	-58.4	2.3	2.1	110.1	114.2	177.4
5OLM_A	CAVHG	-70.7	-35.6	-88.0	-28.3	-111.9	-15.1	-178.1	-61.2	2.3	2.2	108.1	101.0	174.3

6LND_K	CHSHN	-73.0	-17.5	-92.8	-34.2	-103.8	-4.8	173.4	-62.7	2.3	2.1	104.5	106.0	166.7
Average		-63	-26	-91	-34	-116	10	177	-61	2.31	2.16	112	108	168
STD		(6)	(11)	(13)	(12)	(12)	(32)	(6)	(6)	(0.04)	(0.13)	(6)	(7)	(13)
**Cluster 3**														
1WUR _A	CEHHL	-66.4	-18.2	-79.5	-34.9	-127.2	-5.5	71.1	-56.4	2.3	2.2	113.2	105.0	159.0
1WUR_B	CEHHL	-68.7	-17.1	-79.7	-35.6	-127.5	-5.3	72.2	-54.9	2.3	2.3	109.4	105.1	154.2
1WUR_C	CEHHL	-63.0	-21.4	-77.6	-37.1	-124.2	-7.7	71.9	-61.4	2.3	2.4	110.3	104.2	159.6
1WUR_D	CEHHL	-64.5	-18.7	-78.0	-40.5	-122.1	-6.3	71.4	-57.0	2.4	2.2	111.3	104.8	162.5
1WUR_E	CEHHL	-67.2	-18.3	-78.5	-40.3	-122.4	-6.8	65.7	-56.8	2.5	2.2	110.8	100.3	161.7
**3MHJ _A**	**CPVHK**	**-62.5**	**-40.3**	**-74.6**	**-42.5**	**-113.4**	**-5.6**	**66.8**	**-48.1**	**2.3**	**2.1**	**107.7**	**102.6**	**169.1**
3MHJ _B	CPVHK	-66.1	-29.1	-82.0	-45.7	-112.4	-3.6	66.7	-50.6	2.3	2.2	110.2	104.8	172.2
4TOR_A	CPTHK	-62.4	-40.5	-78.8	-43.3	-108.0	-8.1	62.7	-50.4	2.3	2.1	107.9	103.3	165.8
4TOR_B	CPTHK	-58.4	-41.1	-79.9	-43.4	-108.0	-7.3	64.0	-46.5	2.3	2.0	108.4	101.9	173.3
4TOR_C	CPTHK	-58.1	-40.6	-81.5	-43.7	-108.3	-6.9	65.7	-50.6	2.3	2.1	108.1	103.1	169.9
4TOR_D	CPTHK	-60.5	-40.8	-81.3	-42.1	-107.7	-9.3	64.3	-49.7	2.3	2.1	108.5	101.6	170.2
5C5R_A	CPVHK	-59.8	-41.4	-71.3	-43.3	-116.2	-3.5	63.8	-52.8	2.3	2.2	109.7	100.6	168.0
5C5R_B	CPVHK	-64.2	-28.6	-82.5	-41.4	-116.3	-6.6	64.2	-53.6	2.4	2.2	110.3	102.1	164.8
Average		-63	-31	-79	-41	-116	-6	67	-53	2.33	2.16	110	103	165
STD		(3)	(10)	(3)	(3)	(7)	(2)	(3)	(4)	(0.05)	(0.10)	(2)	(2)	(6)
**Cluster 4**														
2V0C_A†	CWRHE	-50.6	-29.3	-94.4	-34.3	-113.6	81.2	174.3	-59.5	2.3	2.2	112.6	104.2	168.8
2YVR_ A†	CNVHK	-81.0	-15.4	-96.8	-49.0	-105.1	83.4	-179.8	-51.7	2.4	2.1	109.5	106.0	168.6
2YVR_ B†	CNVHK	-66.9	-27.0	-96.7	-50.6	-101.1	87.7	-178.2	-52.1	2.4	2.0	111.7	104.7	168.9
**3DDT_A†**	**CKEHE**	**-64.6**	**-35.9**	**-80.8**	**-39.7**	**-117.2**	**86.7**	**-177.1**	**-55.2**	**2.4**	**2.2**	**110.3**	**105.9**	**161.1**

3DDT_B†	CKEHE	-64.1	-44.8	-71.8	-41.9	-114.6	93.5	-178.1	-52.8	2.4	2.0	111.8	100.5	170.0
3DDT_C†	CKEHE	-66.1	-38.2	-84.3	-24.6	-128.4	78.2	-174.2	-60.4	2.3	2.2	115.9	105.4	168.2
3Q1D_A†	CEEHE	-64.4	-39.4	-84.5	-49.0	-98.7	81.1	179.5	-53.7	2.3	2.2	110.3	105.4	169.9
3RYM _A†	CTPHP	-54.5	-51.6	-75.0	-18.4	-130.3	67.5	-175.7	-43.8	2.3	2.1	110.3	102.8	175.8
3RYM_B†	CTPHP	-54.3	-52.6	-74.4	-14.8	-135.7	66.6	-174.6	-45.8	2.2	2.1	111.8	102.0	173.1
3RYM_C†	CTPHP	-54.5	-51.2	-75.6	-19.0	-129.1	67.9	-174.3	-42.3	2.3	2.1	116.3	102.0	170.6
3RYM_D†	CTPHP	-56.5	-50.4	-75.6	-18.9	-129.9	66.7	-175.7	-44.1	2.3	2.1	113.2	102.4	174.9
Average<br>STD		-62<br>(8)	-40<br>(11)	-83<br>(9)	-33<br>(13)	-119<br>(12)	78<br>(9)	-178<br>(3)	-51<br>(6)	2.32<br>(0.04)	2.12<br>(0.08)	112<br>(2)	104<br>(2)	170<br>(4)
**Outlier 5**														
**2AU3_A†**	**CPFHP**	**-85.8**	**-3.1**	**-109.5**	**-24.1**	**-134.9**	**158.0**	**-175.1**	**93.2**	**2.3**	**2.0**	**104.4**	**97.1**	**155.1**
**Outlier 6**														
**5AF0_B**	**CERHE**	**-58.3**	**-47.8**	**-58.7**	**-53.8**	**-63.2**	**-44.6**	**-176.7**	**-81.5**	**2.7**	**2.0**	**95.3**	**99.1**	**137.3**
**Outlier 7**														
**6VHF_A†**	**CKPHN**	**-121.3**	**-36.6**	**-57.2**	**-30.5**	**-114.7**	**18.1**	**178.4**	**-49.6**	**2.4**	**2.2**	**117.5**	**101.8**	**149.5**

[a] The identifier of the chain containing the $C-X_1-X_2-H-X_3$ motif is specified.

† These structures do not show the expected *i, i+4* hydrogen bond (mostly Pro residue in X_3 position).

Table S5. Torsion angles of Zn(II)-METP3 complex as obtained from REM (first row Chain A, second row Chain B) calculations *in vacuo* at 300 K.

Residue	ϕ (°)	ψ (°)	χ^1 (°)
**Cys1**	-119	139	-178
	-133	156	-174
**Thr2**	-74	-21	57
	-96	-3	54
**Gly3**	-86	-8	
	-112	-18	
**His4**	-157	6	88
	-140	8	89
**Ser5**	75	-75	-166
	76	-68	-165
**Gly6**	81	179	
	78	172	
**Asn7**	-118	-165	65
	-116	-167	68
**Aib8**	-41	-44	
	-50	-35	
**Ser9**	-67	-42	73
	-64	-44	80
**Glu10**	-66	-40	-61
	-65	-40	-60
**Ile11**	-72	-57	-57
	-69	-57	-54

Table S6. Intramolecular hydrogen bonds of Zn(II)-METP3 complex as obtained from REM calculations *in vacuo* at 300K.

**Donor (D)**	**Acceptor (A)**	**d $_{(D...A)}$ (Å) chain A**	**d $_{(D...A)}$ (Å) chain B**
Cys^{1}HN	Glu^{10}C'O	2.99	2.99
Ser^{5}HN	Cys^{1}C'O	2.73	2.72
Ile^{11}HN	Gly^{7}C'O	3.20	3.20
Asn^{7}HN	Glu^{10}Oε1	2.87	2.87
His^{4}Nε2	Glu^{10}Oε2	2.83	2.83
Ser^{9}HN	Asn^{7} Oδ1	3.10	3.10
Asn^{7}Nδ	Glu^{10}Oε1	3.02	3.03
Asn^{7}Nδ	Ser^{9}Oγ	3.30	3.30
